# AgBioData consortium recommendations for sustainable genomics and genetics databases for agriculture

**DOI:** 10.1093/database/bay088

**Published:** 2018-09-18

**Authors:** Lisa Harper, Jacqueline Campbell, Ethalinda K S Cannon, Sook Jung, Monica Poelchau, Ramona Walls, Carson Andorf, Elizabeth Arnaud, Tanya Z Berardini, Clayton Birkett, Steve Cannon, James Carson, Bradford Condon, Laurel Cooper, Nathan Dunn, Christine G Elsik, Andrew Farmer, Stephen P Ficklin, David Grant, Emily Grau, Nic Herndon, Zhi-Liang Hu, Jodi Humann, Pankaj Jaiswal, Clement Jonquet, Marie-Angélique Laporte, Pierre Larmande, Gerard Lazo, Fiona McCarthy, Naama Menda, Christopher J Mungall, Monica C Munoz-Torres, Sushma Naithani, Rex Nelson, Daureen Nesdill, Carissa Park, James Reecy, Leonore Reiser, Lacey-Anne Sanderson, Taner Z Sen, Margaret Staton, Sabarinath Subramaniam, Marcela Karey Tello-Ruiz, Victor Unda, Deepak Unni, Liya Wang, Doreen Ware, Jill Wegrzyn, Jason Williams, Margaret Woodhouse, Jing Yu, Doreen Main

**Affiliations:** 1 Corn Insects and Crop Genetics Research Unit, USDA-ARS, Ames, IA, USA; 2 Computer Science, Iowa State University, Ames, IA, USA; 3 Horticulture, Washington State University, Pullman, WA, USA; 4 National Agricultural Library, USDA Agricultural Research Service, Beltsville, MD, USA; 5 Cyverse, University of Arizona, Tucson, AZ, USA; 6 Bioversity International, Informatics Unit, Conservation and Availability Programme, Parc Scientifique Agropolis II, Montpellier, France; 7 The Arabidopsis Information Resource, Phoenix Bioinformatics, Fremont, CA, USA; 8 USDA, Plant, Soil and Nutrition Research, Ithaca, NY, USA; 9 Texas Advanced Computing Center, The University of Texas at Austin, Austin, TX, USA; 10 Entomology and Plant Pathology, University of Tennessee Knoxville, Knoxville, TN, USA; 11 Department of Botany and Plant Pathology, Oregon State University, Corvallis, OR, USA; 12 Environmental Genomics and Systems Biology, Lawrence Berkeley National Laboratory, Berkeley, CA, USA; 13 Division of Animal Sciences and Division of Plant Sciences, University of Missouri, Columbia, MO, USA; 14 National Center for Genome Resources, Santa Fe, NM, USA; 15 Ecology and Evolutionary Biology, University of Connecticut, Storrs, CT, USA; 16 Animal Science, Iowa State University, Ames, USA; 17 Laboratory of Informatics, Robotics, Microelectronics of Montpellier, University of Montpellier & CNRS, Montpellier, France; 18 DIADE, University of Montpellier, IRD, Montpellier, France; 19 Crop Improvement and Genetics Research Unit, USDA-ARS, Albany, CA, USA; 20 School of Animal and Comparative Biomedical Sciences, University of Arizona, Tucson, AZ, USA; 21 Boyce Thompson Institute, Ithaca, NY, USA; 22 Genomics Division, Lawrence Berkeley National Laboratories, Berkeley, CA, USA; 23 Marriott Library, University of Utah, Salt Lake City, UT, USA; 24 Department of Plant Sciences, University of Saskatchewan, Saskatoon, Canada; 25 Plant Genomics, Cold Spring Harbor Laboratory, Cold Spring Harbor, NY, USA; 26 Cold Spring Harbor Laboratory, DNA Learning Center, Cold Spring Harbor, NY, USA; 27 Department of Ecology, Evolution, and Organismal Biology, Iowa State University, Ames, IA, USA

## Abstract

The future of agricultural research depends on data. The sheer volume of agricultural biological data being produced today makes excellent data management essential. Governmental agencies, publishers and science funders require data management plans for publicly funded research. Furthermore, the value of data increases exponentially when they are properly stored, described, integrated and shared, so that they can be easily utilized in future analyses. AgBioData (https://www.agbiodata.org) is a consortium of people working at agricultural biological databases, data archives and knowledgbases who strive to identify common issues in database development, curation and management, with the goal of creating database products that are more Findable, Accessible, Interoperable and Reusable. We strive to promote authentic, detailed, accurate and explicit communication between all parties involved in scientific data. As a step toward this goal, we present the current state of biocuration, ontologies, metadata and persistence, database platforms, programmatic (machine) access to data, communication and sustainability with regard to data curation. Each section describes challenges and opportunities for these topics, along with recommendations and best practices.

## Introduction

We are in an exciting time in Biology. Genomic discovery on a large scale is cheaper, easier and faster than ever. Picture a world where every piece of biological data is available to researchers from easy-to-find and well-organized resources; the data are accurately described and available in an accessible and standard formats; the experimental procedures, samples and time points are all completely documented; and researchers can find answers to any question about the data that they have. Imagine that, with just a few mouse-clicks, you could determine the expression level of any gene under every condition and developmental stage that has ever been tested. You could explore genetic diversity in any gene to find mutations with consequences. Imagine seamless and valid comparisons between experiments from different groups. Picture a research environment where complete documentation of every experimental process is available, and data (with complete metadata) are always submitted to permanent public repositories, where they can be easily found and examined. We ‘can’ imagine that world, and feel strongly that all outcomes of publicly funded research can and should contribute to such a system. It is simply too wasteful to ‘not’ achieve this goal.

Proper data management is a critical aspect of research and publication. Scientists working on federally funded research projects are expected to make research findings publicly available. Data are the lifeblood of research, and their value often do not end with the original study, as they can be reused for further investigation if properly handled. Data become much more valuable when integrated with other data and information (1, 2). For example, traits, images, seed/sample sources, sequencing data and high-throughput phenotyping results become much more informative when integrated with germplasm accessions and pedigree data. Access to low-cost, high-throughput sequencing, large-scale phenotyping and advanced computational algorithms, combined with significant funding by the National Science Foundation (NSF), the US Department of Agriculture (USDA) and the US Department of Energy (DOE) for cyberinfrastructure and agricultural-related research have fueled the growth of databases to manage, store, integrate, analyse and serve these data and tools to scientists and other stakeholders.

To describe agricultural-related databases, we use the term ‘GGB (genomic, genetic and breeding) database’. GGB databases include any online resource that holds genomic, genetic, phenotypic and/or breeding-related information and that is organized via a database schema, and contained within a database management system (or DBMS, which is a computer application that manages and provides access to one or more types of database systems, including relational, Resource Description Framework (RDF), or nonrelational (noSQL) storage systems). GGB databases play a central role in the communities they serve by curating and distributing published data, by facilitating collaborations between scientists and by promoting awareness of what research is being done and by whom in the community. GGB databases prevent duplicated research efforts and foster communication and collaboration between laboratories (2, 3). As more and more organisms are sequenced, cross-species investigations become increasingly informative, requiring researchers to use multiple GGB databases and requiring that GGB databases share data and use compatible software tools. Use of common data standards, vocabularies, ontologies and tools will make curation more effective, promote data sharing and facilitate comparative studies (2).

The AgBioData consortium (https://www.agbiodata.org) was formed in 2015 in response to the need for GGB personnel to work together to come up with better, more efficient database solutions. The mission of the consortium, comprised of members responsible for over 25 GGB databases and allied resources, is to work together to identify ways to consolidate and standardize common GGB database operations to create database products with more interoperability. Member databases and resources are listed at https://www.agbiodata.org/databases and https://fairsharing.org/collection/AgBioData. The AgBioData consortium joins the larger scientific community in embracing the Findable, Accessible Interoperable, and Reusable (FAIR) data principles, established by stakeholders from the scientific, publishing and library communities (4). FAIR principles have rapidly become standard guidelines for proper data management, as they outline a roadmap to maximize data reuse across repositories. However, more specific guidelines on how to implement FAIR principles for agricultural GGB data are needed to assist and streamline implementation across GGB databases.

Members of the AgBioData consortium convened in Salt Lake City, UT on 18 & 19 April 2017 to describe challenges and recommendations for seven topics relevant to GGB databases—**Biocuration, Ontologies, Metadata and persistence, GGB database platforms, Programmatic (Machine) access to data, Communication** and **Sustainability**. Preceding this workshop, a survey was sent out to all AgBioData members regarding the seven topics, in order to identify concerns and challenges of AgBioData members. The results were used to focus and foster the workshop discussions. Here we present the current challenges facing GGBs in each of these seven areas and recommendations for best practices, incorporating discussions from the Salt Lake City meeting and results of the survey.

The purpose of this paper is 3-fold: first, to document the current challenges and opportunities of GGB databases and online resources regarding the collection, integration and provision of data in a standardized way; second, to outline a set of standards and best practices for GGB databases and their curators; and third, to inform policy and decision makers in the federal government, funding agencies, scientific publishers and academic institutions about the growing importance of scientific data curation and management to the research community. The paper is organized by the seven topics discussed at the Salt Lake City workshop. For each topic, we provide an overview, challenges and opportunities and recommendations. The acronym ‘API’ (Application Programming Interface) appears frequently in this paper, referring to the means by which software components communicate with each other: i.e. a set of instructions and data transfer protocols.

We envision this paper will be helpful to scientists in the GGB database community, publishers, funders and policy makers and agricultural scientists who want to broaden their understanding of FAIR data practices.

## Biocuration

### Overview

Biocurators strive to present an accessible, accurate and comprehensive representation of biological knowledge (5–7). Biocuration is the process of selecting and integrating biological knowledge, data and metadata within a structured database so that it can be accessible, understandable and reusable by the research community. Data and metadata are taken from peer-reviewed publications and other sources and integrated with other data to deliver a value-added product to the public for further research. Biocuration is a multidisciplinary effort that involves subject area experts, software developers, bioinformaticians and researchers. The curation process usually includes a mixture of manual, semi-automated and fully automated workflows.

Manual biocuration is the process of an expert reading one or several related publications, assessing and/or validating the quality of the data and entering data manually into a database using curation tools, or by providing spreadsheets to the database manager. It also encompasses the curation of facts or knowledge, in addition to raw data; for example, the role a gene plays in a particular pathway. These data include information on genes, proteins, DNA or RNA sequences, pathways, mutant and non-mutant phenotypes, mutant interactions, qualitative and quantitative traits, genetic variation, diversity and population data, genetic stocks, genetic maps, chromosomal information, genetic markers and any other information from the publication that the curator deems valuable to the database consumers. Manual curation includes determining and attaching appropriate ontology and metadata annotations to data. This sometimes requires interaction with authors to ensure data is represented correctly and completely, and indeed to ask where the data resides if they are not linked to a publication. In well-funded large GGB databases, manually curated data may be reviewed by one, two or even three additional curators.

Manual biocuration is perhaps the best way to curate data, but no GGB database has enough resources to curate all data manually. Moreover, the number of papers produced by each research community continues to grow rapidly. Thus, semi-automated and fully automated workflows are also used by most databases. For example, a species-specific database may want to retrieve all Gene Ontology (GO) annotations for genes and proteins for their species from a multi-species database like UniProt (http://www.uniprot.org). In this case, a script might be written and used to retrieve that data ‘en masse’. Prediction of gene homologs, orthologs and function can also be automated. Some of these standard automated processes require intervention at defined points from expert scientist to choose appropriate references, cut off values, perform verifications and do quality checks. All biocuration aims to add value to data. Harvesting biological data from published literature, linking it to existing data and adding it to a database enables researchers to access the integrated data and use it to advance scientific knowledge.

The manual biocuration of genes, proteins and pathways in one or more species often leads to the development of algorithms and software tools that have wider applications and contribute to automated curation processes. For example, The Arabidopsis Information Resource (TAIR, https://www.arabidopsis.org) has been manually adding GO annotations to thousands of *Arabidopsis* genes from the literature since 1999. This manual GO annotation is now the gold standard reference set for all other plant GO annotations and is used for inferring gene function of related sequences in all other plant species (8–10). Another example is the manually curated metabolic pathways in Ecocyc, MetaCyc and PlantCyc, which have been used to predict genome-scale metabolic networks for several species based on gene sequence similarity (11, 12). The recently developed Plant Reactome database has further streamlined the process of orthology-based projections of plant pathways by creating simultaneous projections for 74 species. These projections are routinely updated along with the curated pathways from the Reactome reference species *Oryza sativa* (13).

Without manual biocuration of experimental data from *Arabidopsis*, rice and other model organisms, the plant community would not have the powerful gene function prediction workflows we have today, nor would the development of the wide array of existing genomic resources and automated protocols have been possible. Biocurators continue to provide feedback to improve automated pipelines for prediction workflows (e.g. genome annotation, mapping etc.) and help to streamline data sets for their communities and/or add a value to the primary data. All biocuration is time consuming and requires assistance from expert biologists. Current efforts in machine learning and automated text mining to pull data or to rank journal articles for curation more effectively work to some extent, but so far these approaches are not able to synthesize a clear narrative and thus cannot yet replace biocurators. The manual curation of literature, genes, proteins, pathways etc. by expert biologists remains the gold standard used for developing and testing text mining tools and other automated workflows. We expect that although text-mining tools will help biocurators achieve higher efficiency, biocurators will remain indispensable to ensure accuracy and relevance of biological data.

Well-curated GGB databases play an important role in the data lifecycle by facilitating dissemination and reuse. GGB databases can increase researchers’ efficiency, increase the return on research funding investment by maximizing reuse and provide use metrics for those who desire to quantify research impact. We anticipate that the demand for biocurators will increase as the tsunami of ‘big data’ continues. Despite the fact that the actual cost of data curation is estimated to be less than 0.1% of the cost of the research that generated primary data (14), data curation remains underfunded (15, 16).

### Challenges and opportunities

#### Biocuration is not keeping pace with research and the generation of data

Databases are focused on serving the varied needs of their stakeholders. Because of this, different GGB databases may curate different data types or curate similar data types to varying depths, and are likely to be duplicating efforts to streamline curation. In addition, limited resources for most GGB databases often prevent timely curation of the rapidly growing data in publications.

#### Researchers use many different databases in their work

We recognize there is a clear need for databases that are tailored for the communities that they serve. For example, SoyBase (https://www.soybase.org) (17) primarily focuses on resources and tools for soybean breeding, while TAIR (18) focuses on gene function for the *Arabidopsis* research community. However, as more organism genomes are sequenced, comparisons between and among species become more biologically informative. Multi-organism databases like Gramene (www.gramene.org) and Phytozome (https://phytozome.jgi.doe.gov), Planteome (www.planteome.org) and European Bioinformatics Institute (EBI)’s gene Expression Atlas (19) etc. provide resources for comparative genomics studies and link data types from various public databases including species-specific databases. This means that researchers now increasingly have access to several different databases and benefit from their cross-referencing to compare data across species. Use of common data standards, vocabularies, ontologies and tools will not only make curation and data sharing more effective, but will also make databases easier to use.

#### Training of data generators in data management skills is poor

Since data management has not traditionally been a part of scientific training, the awareness of the importance of data management is not widespread and the training resources are scarce. As a result, data management by data generators is often insufficient and can result in loss of crucial data as well as the associated metadata. With proper training, and a well-crafted data management plan, researchers can increase the value and reproducibility of their research and ultimately increase the importance of their contribution to science.

#### Community curation is still in its infancy

Meeting the increased need for biocuration will require engagement by the scientific community, however, to date, community curation has not been widely embraced by data generators. Data entry tools where they exist, may be difficult to learn, and graduate students and postdocs may have little motivation or time to curate. To increase community curation at this time, biocurators will need to educate researchers, funders and publishers of the need for publicly sharing accurate research data in reusable forms (20). Crowdsourcing shows promise, but is still new (14, 21).

#### Training for biocurators

We anticipate that the demand for biocurators will increase as the tsunami of ‘big data’ continues. Biocurators need a certain set of skills in both computational methods and biological expertise in their chosen area. The University of Cambridge has just launched a new program leading to a Postgraduate Certificate in Biocuration (https://www.ice.cam.ac.uk/course/postgraduate-certificate-biocuration), but there are no graduate programs in biocuration that we are aware of.

### Recommendations

#### Developing AgBioData standards of curation

One reason for establishing the AgBioData group was to provide a forum to share curation methods from different GGB databases in order to improve and streamline the process. Although the demand for data curation accelerates while resources for biocuration remain limited, by working together, we reduce redundancy and continually improve our curation methods. Each GGB database is responsible for selecting data and providing consistent curation based on the needs of its users. However, the use of curation standards will increase consistency across databases and promote efficiency and interoperability. To move toward more standard biocuration methods, we recommend the following practices:

1. **Continue regular communication between biocurators of different databases**. AgBioData holds monthly conference calls that frequently include discussions on curation issues or reports on biocuration projects from within the AgBioData group and from outside groups. AgBioData also encourages member participation and interaction with the broader (non-agricultural) biocuration community, such as with the International Society for Biocuration.
2. **Adopt existing or contribute to developing new Minimum Information for Biological and Biomedical Investigations recommendations for each curated data type.** As minimal data and metadata recommendations are established, AgBioData will provide this information to researchers, funders and publishers through publications and seminars, and will strongly encourage their consistent use. These recommendations will also be provided via appropriate online resources such as AgBioData.org and FAIRsharing.org.
3. **Share data curation templates.** Many biocurators develop and use template spreadsheets for data collection from literature. At MaizeGDB (22), for example, templates have been used to ensure consistency between curators and to ease loading of curated data. Database biocurators who have expertise in curation of certain data types are encouraged to share their data templates and curation guidelines through AgBioData.org. These expert templates and guidelines can be adopted for use by other GGB databases as needed. Sharing templates will also support communities in the process of establishing a new database, as they will not have to reinvent curation best practices.
4. **Recommend that funding agencies require data and metadata submission to public resources as a requirement for funding.** Funding agencies should hold grantees accountable by requiring proof of proper data submissions to a public resource. GGB databases can help by including funding sources and grant IDs in the metadata.
5. **Recommend that publishers require data and metadata submission to public resource as a requirement for publication**. Publishers should require authors to submit to archival resources and require proof of proper data submission to public resources before publication. This was very successful in the early stages of establishing the submission of germplasm to the Arabidopsis Biological Resource Center or requiring GenBank IDs for any sequence. GGB databases can help by providing best practices and support for ingestion of these data sets.
6. **Databases should encourage and facilitate use of persistent digital identifiers by data generators.** Consistent use of unique Digital Object Identifiers (DOIs) or Archival Resource Keys (ARKs) will reduce errors in referring to data sets within and across different GGB databases.
7. **Report errors and omissions of data to authors, editors, databases and publishers.** A reporting system that curators can use to notify the authors and journal editors when a paper with missing data or metadata is discovered will help ensure proper data dissemination. Encouraging researchers to provide feedback to GGB databases when they find errors or omissions is important as well.
8. **Work toward increasing the visibility of biocurators and what they contribute to the research community.** Many scientists do not know the amount of work, and manual curation, that is needed to maintain a community database. Educating the users of community GGB databases, and the people behind them, could go a long way to encourage proper data submission.
9. **Provide training for researchers on responsible data management.** Tutorials covering all aspects of data management, including file formats, the collection and publishing of high value metadata along with data, interacting with GGB databases, how to attach a license to your data, how to ensure your data stays with your publication and more will be useful training tools. This information can be presented as short online videos or FAQs and as live presentations to researchers. Encouraging collaborations between biocurators and educators to integrate data management and stewardship strategies into graduate and undergraduate course materials allows for the training of the next generation of scientists in responsible data management practices. Course work of this type can be developed in collaboration with institutional librarians who have a similar mission to provide training in data stewardship.
10. **Provide post-graduate training in biocuration.** Biocurators need both computation skills and biological expertise. We strongly encourage development of programs for biocurators and the training of graduate students in biocuration.

## Ontologies

### Overview

The size and the complexity of biological data resulting from recent technological advances require the data to be stored in computable or standardized form for efficient integration and retrieval. Use of ontologies to annotate data is important for integrating disparate data sets. Ontologies are structured, controlled vocabularies (CVs) that represent specific knowledge domains (23). Examples include the GO (24) for attributes of gene products such as subcellular localization, molecular function or biological role, and Plant Ontology (PO) (25–27) for plant attributes such as developmental stages or anatomical parts. When data are associated with appropriate ontology terms, data interoperability, retrieval and transfer are more effective. In this section, we review the challenges and opportunities in the use of ontologies and provide a set of recommendations for data curation with ontologies.

### Challenges and opportunities

To identify current status and challenges in ontology use, an online survey was offered to AgBioData members. The survey results for ontology use in databases for each data type are provided in Table 1 and a summary of other survey questions such as barriers to using ontologies are provided in the supplementary material 1. In addition, the ways ontologies are used in data descriptions in some GGB databases are described in supplementary material 2.

**Table 1:** Survey results for ontology use in databases for each data type (from 29 respondents)

	Sequence (27)	Marker (18)	QTL (15)	Germplasm (16)	Phenotype (20)	Genotype (18)
GO	17	1	2			1
SO	10	1				2
PO	4	2	2	2	1	
Trait ontologies: TO/VT/LPT	1	1	7	2	3	
CO			1		3	
Other ref ontology	1 (MI)		1 (LBO, CMO)	1	1 PATO	
In-house			1	3		

**Table 2:** List of ontologies, CVs and thesaurus of interest for AgBioData member databases

Name	Domain	ID space	URL
Amphibian Gross Anatomy Ontology	Amphibian anatomy	AAO	http://purl.bioontology.org/ontology/AAO
Agronomy Ontology	agronomy trials	AGRO	http://obofoundry.org/ontology/agro.html
AGROVOC	a controlled vocabulary covering all areas of interest of the Food and Agriculture Organization	AGROVOC	http://aims.fao.org/vest-registry/vocabularies/agrovoc-multilingual-agricultural-thesaurus
Animal Trait Ontology for Livestock	phenotypic traits of farm animals	ATOL	http://purl.bioontology.org/ontology/ATOL
CAB Thesaurus	bibliographic databases of CABI (Centre for Agriculture and Biosciences International)	CABT	http://www.cabi.org/cabthesaurus/
Cephalopod Ontology	cephalopod anatomy and development	CEPH	http://obofoundry.org/ontology/ceph.html
Chemical Entities of Biological Interest	molecular entities	CHEBI	http://obofoundry.org/ontology/chebi.html
Cell Ontology	Metazoan (not plant) cell types	CL	http://obofoundry.org/ontology/cl.html
Clinical Measurement Ontology	morphological and physiological measurement records generated from clinical and model organism research and health programs	CMO	http://obofoundry.org/ontology/cmo.html
Crop Ontology	a collection of vocabularies that describe breeders’ traits for agriculturally important plants: banana, barley, beets, Brachiaria, brassica, cassava, castor bean, chickpea, common bean, cowpea, grapes, groundnut, lentil, maize, mung bean, pearl millet, pigeon pea, potato, rice, sorghum, soybean, sugar kelp, sweet potato, wheat, woody plant and yam	CO	http://www.cropontology.org/
Drosophila Phenotype Ontology	Drosophila phenotypes	DPO	http://obofoundry.org/ontology/dpo.html
Evidence and Conclusion Ontology	types of scientific evidence	ECO	http://www.evidenceontology.org/Welcome.html http://obofoundry.org/ontology/eco.html
Experimental Factor Ontology	anatomy, disease and chemical compounds	EFO	http://www.ebi.ac.uk/efo/
Environment Ontology	biomes, environmental features and environmental materials	ENVO	http://obofoundry.org/ontology/envo.html
Feature Annotation Location Description Ontology	FALDO is the Feature Annotation Location Description Ontology. It is a simple ontology to describe sequence feature positions and regions as found in GFF3, DBBJ, EMBL, GenBank files, UniProt and many other bioinformatics resources.	FALDO	https://github.com/JervenBolleman/FALDO
Drosophila Gross Anatomy Ontology	Drosophila melanogaster anatomy	FB-BT	http://obofoundry.org/ontology/fbbt.html
Flora Phenotype Ontology	traits and phenotypes of flowering plants occurring in digitized floras	FLOPO	http://obofoundry.org/ontology/flopo.html
Gene Ontology	gene function, biological processes and cellular components	GO	http://obofoundry.org/ontology/go.html
Hymenoptera Anatomy Ontology	anatomy of Hymenoptera	HAO	http://hymao.org http://obofoundry.org/ontology/hao.html
Infectious Disease Ontology	infectious diseases	IDO	http://infectiousdiseaseontology.org/page/Download
Dengue fever	disease ontology for Dengue fever	IDODEN	VectorBase Ontologies
Malaria	disease ontology for malaria	IDOMAL	VectorBase Ontologies
Livestock Breed Ontology	buffalo, cattle, chicken, goat, horse, pig and sheep breeds	LBO	http://purl.bioontology.org/ontology/LBO
Livestock Product trait Ontology	traits of products from agricultural animals or birds	LPT	http://purl.bioontology.org/ontology/LPT
Mammalian Feeding Muscle Ontology	an anatomy ontology for the muscles of the head and neck that participate in feeding, swallowing and other oral-pharyngeal behaviors	MFMO	http://obofoundry.org/ontology/mfmo.html
Molecular interactions	protein–protein interactions	MI	http://www.obofoundry.org/ontology/mi.html
Mosquito Insecticide Resistance	mosquito insecticide resistance	MIRO	VectorBase Ontologies
MONDO Disease Ontology	diseases (currently mostly human but also animal diseases)	MONDO	http://obofoundry.org/ontology/mondo.html
Mammalian phenotype	mammalian phenotypes	MP	http://obofoundry.org/ontology/mp.html
Mouse Pathology Ontology	mutant and transgenic mouse pathological lesions and processes	MPATH	http://obofoundry.org/ontology/mpath.html
National Agricultural Library Thesaurus	vocabulary tools of agricultural terms	NALT	https://agclass.nal.usda.gov/
Neuro Behavior Ontology	behavior terms	NBO	http://www.obofoundry.org/ontology/nbo.html
Ontology of Arthropod Circulatory Systems	arthropod circulatory system	OARCS	http://obofoundry.org/ontology/oarcs.html
Ontology of Biological Attributes	traits (all species)	OBA	http://obofoundry.org/ontology/oba.html
Ontology of Host-Microbe interactions	host–microbe interactions	OHMI	http://www.obofoundry.org/ontology/ohmi.html
Ontology of Microbial Phenotypes	microbial phenotypes	OMP	http://obofoundry.org/ontology/omp.html
Ontology for Parasite Lifecycle	parasite life cycle stages	OPL	http://www.obofoundry.org/ontology/opl.html
Phenotype and Trait Ontology	phenotypic qualities	PATO	http://obofoundry.org/ontology/pato.html
Population and Community Ontology	populations and communities	PCO	http://obofoundry.org/ontology/pco.html
Plant Experimental Conditions Ontology	plant treatments, growing conditions and/or study types	PECO	http://browser.planteome.org/amigo http://obofoundry.org/ontology/peco.html
Plant Ontology	plant anatomy and growth stages	PO	http://browser.planteome.org/amigo http://obofoundry.org/ontology/po.html
Protein Ontology	protein-related entities	PR	http://obofoundry.org/ontology/pr.html
Social Insect Behavior Ontology	chemical, anatomy and behavior of social insects	SIBO	http://www.obofoundry.org/ontology/sibo.html
Sequence Ontology	sequence types and features	SO	http://obofoundry.org/ontology/so.html
SOY Ontology	soybean traits, growth and development	SOY	https://www.soybase.org/ontology.php
Spider anatomy and behavior ontology	spider anatomy, behavior and products	SPD	http://obofoundry.org/ontology/spd.html
Tick anatomy	Tick gross anatomy	TADS	VectorBase Ontologies
Mosquito anatomy	Mosquito gross anatomy	TGMA	VectorBase Ontologies
Plant Trait Ontology	plant traits	TO	http://browser.planteome.org/amigo http://obofoundry.org/ontology/to.html
Tribolium Ontology	anatomy of the red flour beetle *Tribolium castaneum*	TRON	http://ibeetle-base.uni-goettingen.de/ontology/overview.jsf
Teleost Taxonomy Ontology	Teleost phenotypes specifically for zebrafish	TTO	http://obofoundry.org/ontology/tto.html
Uberon multispecies anatomy ontology	animal anatomical structures	Uberon	http://obofoundry.org/ontology/uberon.html
Variation Ontology	variations in DNA, RNA and/or protein	VARIO	http://purl.obolibrary.org/obo/vario.owl
VectorBase controlled vocabulary	controlled vocabulary for vector biology	VBCV	VectorBase Ontologies
Vertebrate Trait Ontology	morphology, physiology or development of vertebrates	VT	http://obofoundry.org/ontology/vt.html
Xenopus anatomy and development ontology	anatomy and development of Xenopus sp.	XAO	http://www.xenbase.org/anatomy/xao.do?method=display http://obofoundry.org/ontology/xao.html
Zebrafish Anatomy and Development Ontology	Zebrafish anatomy and development	ZFA	http://zfin.org/zf_info/anatomy/dict/sum.html http://obofoundry.org/ontology/zfa.html
Zebrafish Developmental Stages	Zebrafish (Danio rerio) developmental stages	ZFS	http://obofoundry.org/ontology/zfs.html

To facilitate the adoption of ontologies by GGB databases, we describe the challenges identified by the survey along with some opportunities to meet these challenges, including a review of currently available ontologies for agriculture, ontology libraries and registries and tools for working with ontologies.

#### Ontology annotations in most GGB databases are limited to certain ontologies

Most GGB databases use GO (24) but fewer use additional ontologies such as Plant Trait Ontology (TO) and PO (25–27) to describe their data. In addition, with a few exceptions, these terms are assigned through computation instead of through rigorous manual annotation. The use of ontologies could be facilitated if the list of applicable ontologies were readily available. Within the agricultural domain there are many reference ontologies applicable to model and crop plants, livestock, arthropods and other animal species. Table 2 lists some of the ontologies that are applicable to agricultural data. In supplementary material 3, we also describe ontology libraries and registries, including description of the Planteome project (http://planteome.org), the Crop Ontology project (CO) (www.cropontology.org) (28), the Open Biological and Biomedical Ontology (OBO) Foundry (http://www.obofoundry.org) (29), the NCBO BioPortal (http://bioportal.bioontology.org) (30), OntoBee (http://www.ontobee.org) (31), the EBI Ontology Lookup Service (http://www.ebi.ac.uk/ols) (32), AberOWL (http://aber-owl.net) (33) and the AgroPortal project (http://agroportal.lirmm.fr) (34).

#### Lack of funding and resources to train and pay biocurators

While most databases recognize the importance of using ontologies for efficient data integration and retrieval, GGB databases typically lack sufficient funding to train and pay additional biocurators. The curation work could be somewhat eased by tools for the curation and validation of annotations and by standardized data formats for ontology annotation data exchange. Significant work has been done in these areas. The curation tools for GO annotation include TAIR’s in-house curation tool PubSearch (35) and TAIR’s community portal the Online Annotation Submission Tool (TOAST) (18), PomBase’s Canto (36), the GO consortium’s Noctua (http://noctua.berkeleybop.org) and Table Editor (http://bit.ly/table-editor) (Table 3). To facilitate sharing annotations among resources, there are some existing and emergent standards for ontology annotation data exchange. The GO Annotation File (GAF) format is the standard for GO annotation data exchange (http://geneontology.org/page/go-annotation-file-format-20) and Phenopackets (Phenotype Exchange Format or PFX; https://github.com/phenopackets/phenopacket-format/wiki/Overview-of-Phenotype-Exchange-Format) is an extensible data model and data exchange format for phenotype data from any species (Table 3). More details about these tools are provided in supplementary material 4.

**Table 3:** List of tools for data curation with ontologies, annotation data exchange format and tools for ontology editing

Use	Tool	Summary	Reference/URL
Data curation/annotation	Noctua	web-based tool for collaborative editing of models of biological processes	http://noctua.berkeleybop.org/
	PubSearch	TAIR in-house literature curation tool	https://www.ncbi.nlm.nih.gov/pubmed/18428773
	Protein2GO	EBI’s GO annotation tool	https://sourceforge.net/projects/protein2go/
	TOAST	community curation tool for GO and PO annotations	https://www.ncbi.nlm.nih.gov/pubmed/22859749
	CANTO	web-based literature curation tool	https://academic.oup.com/bioinformatics/article/30/12/1791/382357/Canto-an-online-tool-for-community-literature
	Textpresso Central	web-based text mining and literature curation (with plug ins for Noctua)	doi: 10.1186/s12859-018-2103-8.
	CACAO	community annotation tool used in undergraduate competitions	https://gowiki.tamu.edu/wiki/index.php/Category:CACAO
	Table Editor	application for easily editing spreadsheet-formatted data with associated ontologies	https://incatools.github.io/table-editor/?config=.%2Fconfig.yaml
	PhenoteFX	phenotype curation	http://phenotefx.readthedocs.io/en/latest/
Annotation data exchange formats	GAF2	file format for ontology annotation data exchange	http://geneontology.org/page/go-annotation-file-format-20
	RDF	Resource Description Framework	
	Phenopackets	an extensible data model and data exchange format for phenotype data	https://github.com/phenopackets/phenopacket-format/wiki/Overview-of-Phenotype-Exchange-Format
	BioLink model	schema for biological data and associations	https://biolink.github.io/biolink-model/
Ontology editors	Protégé	ontology editing tool	http://protege.stanford.edu/

#### Lack of ontologies that fit the domain or insufficient terms in existing ontologies

Some databases develop in-house ontologies since the existing ontologies do not meet their needs. When using in-house ontologies, it is necessary to map their terms to reference ontologies to facilitate ontology development and/or data transfer among other databases. In addition, it is often necessary to use species-specific ontologies. For example, analogous organs across plant species often do not have the same name. To ease this difficulty, the CO and Planteome projects work together to link terms in plant species-specific ontologies to more general terms in references ontologies like GO and PO. In case of incomplete ontologies, there is a need for a tool or system where researchers and biocurators can add terms, which are timely reviewed for inclusion in the ontologies.

### Recommendations

Based on the challenges we identified, we provide the following recommendations.

#### Use ontologies in data curation

The core recommended set of ontologies to use for agricultural is GO for gene function annotation, Sequence Ontology (SO) to define sequence types and trait ontologies for Quantitative trait locus (QTL), heritable phenotypic markers, gene models, transcripts, germplasm, molecular markers and trait descriptors for breeding and/or diversity projects.

PO and TO are recommended for describing plant anatomy, developmental stages of plants and plant traits. When species-specific trait ontologies are used, it is recommended that they be annotated with reference PO and TO to enable cross-species comparison. Trait-related ontologies recommended for livestock, arthropods and other animal species are summarized in Table 4. All curatorial assignments of an ontology term to a database object should also be accompanied with the appropriate Evidence and Conclusions Ontology (ECO) term describing the evidence on which that assertion is based and a traceable reference to the source of the experimental data.

**Table 4:** Trait-related ontologies for describing data from livestock, arthropods and other animals. A key to the ontologies is available in Table 2

Data type	Domain	Ontology (see Table 2)
Phenotype	cattle, sheep, goats, pig other animals	ATOL, LBO, LPT, VT MP, VT, ATOL, ABO
Anatomy	cattle, sheep, goats, pig other animals arthropods	UBERON, CARO XAO, ZFA, CARO, CEPH, MFMO, TTO, UBERON DPO, FBBT, HAO, OARCS, SPD, TGMA, AAO, TRON
Growth and development	cattle, sheep, goats, pig other animals arthropods	ATOL, VT ZFS, CEPH, ATOL FDdv
Behavior	livestock/other animals arthropods	NBO, ATOL, VT SIBO
Disease	growth and development related disease other disease	IDO, OHMI, OPLMPATH, OMP, MONDO

For agricultural animals, anatomy is represented using Uberon (37) and Cell Ontology (CL) (38) (for gross anatomical structures and cell types, respectively). For describing traits, the Vertebrate Trait Ontology (VT) provides trait descriptors analogous to TO for plants. The Ontology of Biological Attributes (OBA) is a Phenotype and Trait Ontology (PATO)-based ontology that provides traits in a completely species-neutral way, and integrates with VT and TO. The Mammalian Phenotype Ontology describes abnormal effects of gene mutations and other phenotypes.

#### Make methods transparent to researchers when computational annotation is done instead of manual curation

Manual and rigorous ontology annotation is recommended. When manual curation is not practical due to lack of curation time, we recommend computational approaches for automatic association of GO or PO terms using the rigorously curated ontology associations from ‘Model’ species based on previously established gene orthology relationships. The Ensembl project (39) produces orthology relationships among a number of plant species in Ensembl-plant (http://plants.ensembl.org) as does the InParanoid project (http://inparanoid.sbc.su.se). Gene family groupings can also be used to infer orthology. A number of gene families have been produced by various groups such as Panther (pantherdb.org) and Pfam (http://pfam.xfam.org). Once gene families are developed for a species vis-à-vis an appropriate model organism, these families, along with GAFs could be used to transfer GO as well as TO annotations between GGB databases with modest curatorial resources by simple identifier matching algorithms. As is common practice, sequence similarity to gene models from model organisms can also be used in GO annotation. It is important, however, to check the computational method and make it transparent to the users.

#### Make an effort to create a network of ontologies as a community

In order to provide unified access to different types of agricultural data and enable large-scale analysis, it is crucial to have a network of domain ontologies. Each ontology focuses on its own domain, but often several ontologies are needed to fully describe the data. The OBO Foundry initiative (29) provides a variety of tools including an ontology import feature. As an example of the creation of an ontology network, TO defines phenotypic traits in plants as Entity–Quality (EQ) statements. The quality (Q) terms come from the PATO whereas the entity (E) terms come from PO, GO or ChEBI, depending on the entity. These efforts can reduce the curation time in individual databases since once the data is curated with one ontology such as TO, it can be further associated with other component ontologies. There are also tools to make relationships among reference ontologies. One example is Intelligent Concept Assistant (https://github.com/INCATools/intelligent-concept-assistant), a National Institute of Health (NIH) Big Data 2 Knowledge (https://commonfund.nih.gov/bd2k)-funded project to develop an environment for helping scientists to collaboratively create, extend and apply their knowledge to describe and interpret biomedical data sets.

#### Facilitate ontology annotation by data generators

We encourage the use of ontologies by implementing rules and procedures where available/applicable, and improving ontologies by enlisting community helps in adding new terms, correcting existing terms as needed and in general, modifying ontologies to be broadly adaptive. A good example of this process occurs in AnimalQTLdb, where ontologies were developed in parallel with improvements to AnimalQTLdb (40).

One way to encourage researchers to use ontologies is to provide a system that requires collection of accepted ontology terms as part of the data and/or publication submission process. Some databases, such as TAIR, TreeGenes (41) and GDR work with journals to require that authors submit their data to the appropriate GGB database prior to manuscript submission (20). There are multiple approaches to this. GDR has downloadable data templates that researchers fill in and submit. TreeGenes has a web form for submitting association genetics and population genomics studies. TreeGene’s web form is currently being converted to a Tripal module (42), with the aim of fully adopting Minimum Information About a Plant Phenotyping Experiment (43) to include the full spectrum of data generation, including design, sampling, sequencing and informatic analysis. The TreeGene system simplifies the submission process through the use of guided prompts to query researcher for the location of the experiment (i.e. latitude, longitude, altitude), the type of experiment (e.g. common garden, reciprocal transplant) and environmental conditions (e.g. average temperature), and to determine which ontologies are necessary. TAIR’s TOAST (18; https://toast.arabidopsis.org/) allows authors to submit GO and PO annotations for their own or for others’ published works.

## Metadata and persistence

### Overview

Public data is valuable for additional research and for reproducibility analyses. But data cannot be reused unless they are sufficiently described, including attribution, analysis methods, procedures, data formats and a description of the subjects and treatments. Data cannot be reused if they cannot be found via search engines or persistent identifiers.

Take the data resulting from a Genome Wide Association Study (GWAS) as an example. The accompanying metadata should include the species and specific individuals that were sampled; the study participants and publication; the genotypes and phenotypes and how they are obtained; the name, versions and parameters of software used; any scripts developed; parameters used to define significance; and data formats. Not only does this enable researchers to reuse data that may have been produced at considerable expense, but also enables researchers to reproduce results (a particular matter of concern in recent years, given the need for improving trust in the scientific process). Furthermore, having a persistent identifier attached to this data set, and having it deposited in a permanent repository, ensures that it can be found, retrieved and reused by multiple researchers for years to come.

Metadata is descriptive information about an object or resource whether it be physical or electronic. The underlying concepts behind metadata have been in use for as long as collections of information have been organized. Library card catalogs represent a well-established type of metadata that have served as collection management and resource discovery tools for decades.

A metadata standard can be either a set of core descriptors that will apply in all instances and extended as needed or a comprehensive standard consisting of both required and optional data fields. The advantage of a core set is that its simplicity can greatly aid its adoption. The Dublin Core Metadata Initiative (44, 45) is an example of a core standard. For description of data sets, a more comprehensive standard would be https://www.w3.org/TR/hcls-dataset/. For scientific research data, a ‘core standard’ will not be adequate to describe how the data was generated and analysed. Extensions to a ‘core standard’ are by definition, not standardized and so, extended fields likely cannot be compared. A ‘comprehensive standard’, on the other hand, may provide sufficient descriptive fields to enable reuse of research data, but its complexity may create a significant barrier to adoption. Another dimension of a metadata standard is the use of CVs. To compare metadata for multiple data sets, there must be a means of directly comparing the contents of each field. CVs, especially in the form of a hierarchical ontology that contains information about the relatedness of values, are essential to metadata.

Metadata is critical to enabling each of the four FAIR principles. Additionally, metadata can be viewed as serving multiple purposes:

- **Administration** - Acquisition, provenance and licensing.
- **Description -** Identify and describe.
- **Preservation** - Long-term management of persistence and relevance, adaptation to changes in technologies, support of old data formats or conversion into new and continued integration with new data.
- **Technical** - Hardware & software documentation, including versions and parameters used in analyses, data formats and versions.

There are multiple audiences for both the production and consumption of metadata:

- **Researchers**: produce: to get credit, consume: to reuse
- **Repositories**: produce: policies for metadata, curate data sets, attach metadata to data set, consume: use metadata in search utilities
- **Journals**: produce: metadata policies
- **Funding agencies**: produce: metadata policies

Metadata is not a new concept and its challenges are well understood and so, has been addressed by a number of disciplines, groups and resources, such as those listed below.

#### FAIRsharing

(formerly BIOsharing; https://fairsharing.org) (46–48) is a UK-based clearinghouse of standards, policies and databases in the life sciences, including the biology and biomedical disciplines. Information about standards, policies and databases is curated by both FAIRsharing staff and community members. The data can be searched by keyword, browsed by type or discovered by way of collections of related resources. An example of a collection is all databases and metadata standards associated with metabolic data. The AgBioData group has started collaborating with the FAIRsharing group, as described below, and is represented at FAIRsharing as an organizational collection (https://fairsharing.org/collection/AgBioData). Member GGB databases are encouraged to ensure that their FAIRsharing records are complete and up to date.

#### Academic libraries

A resource that is close at hand for many researchers and repositories is an academic library. Library science provided one of the earliest metadata standards for digital objects in 1995: the Dublin Core (http://dublincore.org), which remains a foundational recommendation for all types of digital objects. Most academic libraries have one or more data management librarians. To help researchers comply with funding agency policies for data management, data librarians had developed a number of resources:

- Scholarly Publishing and Academic Resources Coalition (SPARC; https://sparcopen.org) and Johns Hopkins University Libraries developed and maintain a listing of the data sharing requirements by federal agencies at http://researchsharing.sparcopen.org.
- Data Management Planning Tool (DMPTool; https://dmptool.org) was developed for writing required data management plans by the California Digital Library and the Data Observation Network for Earth (https://www.dataone.org) project.
- REgistry of Research Data REpositories (Re3Data; http://www.re3data.org) is a database of available repositories for research data developed by the merger of two similar efforts, one in the EU and one in the US.
- The Directory at the Digital Curation Centre (DCC; http://www.dcc.ac.uk) is a good resource for developing data management plans.
- The Metadata Standards Directory Working Group (http://rd-alliance.github.io/metadata-directory), a section of the Research Data Alliance (RDA; https://www.rd-alliance.org), now maintains a listing of metadata standards and their extensions.
- The Ag Data Commons (https://data.nal.usda.gov) is an agriculture-specific registry of databases, data sets and tools with USDA funding support. Its metadata are interoperable with the World Wide Web Consortium (W3C) Data Catalog vocabulary (https://www.w3.org/TR/vocab-dcat/) and the open government Project Open Data v1.1 standard (https://project-open-data.cio.gov/v1.1/schema/).
- Research Data Management Service Group at Cornell University (https://data.research.cornell.edu/content/readme) have developed a guide for writing README files, a frequent form of metadata.
- The Agriculture Network Information Collaborative alliance includes a working group that coordinates data management plan guidance across its member libraries.

#### The RDA

(https://www.rd-alliance.org) was started in 2013 by the European Commission (EC), the NSF, the National Institute of Standards and Technology and the Australian Government's Department of Innovation to encourage and enable open sharing of research data in all scientific disciplines and the humanities. Several RDA working groups are focused on metadata. The RDA Metadata Standards Directory and the Metadata Catalog of metadata standards that have been submitted to the RDA is in progress and can be seen at http://rd-alliance.github.io/metadata-directory.

#### The EC–US workshop on Plant Bioinformatics (2009)

The workshop included representatives from USDA, NIH, DOE, Office of Science and Technology Policy, the Environmental Protection Agency and Food and Drug Administration. One outcome of the workshop was a set of recommendations for international collaboration on education, standards, cyberinfrastructure and stewardship. The recommendations call for a concerted and joint effort to develop standards and ontologies, and the annotation of primary and derived data with descriptive standard metadata.

#### ISA - Investigation, Study, Assay

(http://isa-tools.org/) (49) approach to metadata is structured with three elements: the Investigation—the research context; a Study—a unit of research; and an Assay—an analytical measurement. These data can be provided in a number of formats, tabular, JavaScript Object Notation and RDF. A common representation is ISA-TAB, a tab-delimited format, which is supported by a number of tools (http://isa-tools.org).

#### Collaborative Open Plant Omics (COPO)

(http://copo-project.org) is a UK-funded project to aid deposit of researcher data into appropriate repositories along with the recommended metadata and the discovery and retrieval of public data for reuse. The project has been underway for 3 years and its data submission portal is in beta testing. The portal will support a number of public repositories and will interact with European Nucleotide Archive (ENA), the European member of the International Nucleotide Sequence Database Collaboration (INSDC).

#### transPLANT

( http://www.transplantdb.eu) is a consortium of 11 European partners (http://www.transplantdb.eu/partners) gathered to address the challenges of working with plant genomes and to develop a transnational infrastructure for plant genomic science. Among other objectives, transPLANT aims to develop and share standards for the description of data and metadata, which are critical to allow sharing of information between different resources. One example of this effort is the recommendations for plant phenotyping metadata and data handling put forth in a recent publication (50, 51).

#### Schema.org

(schema.org) is a collaborative effort to improve data set findability by exposing metadata to standard web search engines.

#### INSDC

is a consortium of sequence databases that includes GenBank from National Center for Biotechology Information (NCBI), ENA and DNA Data Bank of Japan. This consortium participated in setting policies for collection of standard metadata and providing online forms and metadata templates for submission of metadata with genomic data sets.

#### MIQAS

(Minimum Information for QTLs and Association Studies) is a collaborative effort between Iowa State University and the Roslin Institute of the University of Edinburgh to standardize the minimum information required for database entry and subsequently to facilitate meaningful meta-analyses of QTL/association results. To date, the effort has produced the following outcomes:

- A website that describes the MIQAS (http://miqas.sourceforge.net),
- Demonstrated application of the required minimum information standards for livestock QTL/GWAS data in Animal QTLdb (https://www.animalgenome.org/QTLdb/doc/minfo; 40),
- Some meta-analyses that benefited from the minimum information framework (52, 53).

#### The Monarch Initiative

(https://monarchinitiative.org) integrates genotype and phenotype data from multiple species and sources. The resulting integrated knowledge graph, analytic tools and web services enable researchers to explore relationships between phenotypes and genotypes across species. Standardized metadata and formal ontologies are used to better enable data sharing, reproducibility and integration. Computer-readable data is shared using the Biolink API (https://github.com/biolink; https://api.monarchinitiative.org/api). Monarch also uses Phenopackets (https://github.com/phenopackets) for standardized data exchange of phenotype data.

#### Genomic Standards Consortium

(http://gensc.org) is an international consortium for developing metadata standards for describing genome data. The consortium includes representatives from many of the groups above and INSDC databases.

#### Genomes Online Database

(GOLD, https://gold.jgi.doe.gov; 54) is an example of extensive use of a metadata standard to enable finding data sets. GOLD holds Minimum Information about any(x) Sequence (http://gensc.org/mixs/) metadata to enable fine-grained searching of sequence data. The sequence data itself is maintained elsewhere, typically in an INSDC.

### Challenges and opportunities

For all the importance of metadata, there are several explanations for why it is often neglected.

- Development of metadata standards is very difficult, requiring consensus and agreement across multiple groups, and requires a governing body to drive the process, manage the standards and encourage or enforce adoption.
- Collection and curation of adequate metadata is also difficult and time consuming for both data curators and researchers.
- Researchers often cannot see the value of thorough metadata and therefore are unwilling to take the time to provide it, or assume the paper ‘is’ the metadata.
- Understaffed repositories may make metadata collection a low priority due to its effort and perceived lack of value.
- Finally, even if a standard exists and a researcher or repository is willing to provide metadata, the appropriate standards can be difficult to find and are often unwieldy; many of the data fields are likely to be irrelevant to a particular data set, repository or study type. Often custom metadata is used, which, although better than no metadata, make it difficult or impossible to search across or compare data sets.

Another challenge is how to permanently attach metadata to data sets to make them discoverable. Too often the meaning of files is implicit in a directory structure, e.g. version number, which is typically lost when files are shared. README files can go missing, file names are changed or provenance is mentioned in a long ago deleted e-mail. This implies that not only is data persistence important but its metadata must also be persistent and permanently attached. Examples of persistent identifiers attached to metadata are GenBank's BioProject and BioSample records that describe genomic, genotypic and expression data deposited at GenBank (55). Also, DOIs (see https://www.doi.org/index.html) and ARKs (see http://n2t.net/e/ark_ids.html) are associated with defined metadata (56). A recent paper (57) and related blog post (http://blogs.plos.org/biologue/2017/07/06/bad-identifiers-potholes-of-information-superhighway/) emphasize the importance of choosing unique identifiers for data sets and for objects within data sets.

### Recommendations

Our main concerns for metadata are the following: use of persistent identifiers; encouraging and enforcing the use of standard and effective metadata; proliferation and duplication of standards development, including frequent use of custom metadata that can't be compared outside a specific GGB database or data set; the difficulty of choosing among large numbers of recommendations and creating profiles to meet specific needs; and the difficulty of filling in extensive metadata forms. To address these concerns, we recommend that researchers and GGB database curators:

#### Work with librarians

at local institutions for training and to help develop data management plans that include collection of appropriate metadata.

#### Curate data and metadata

Manual curation is vital for GGB databases. Without curation, data sets cannot be integrated or interoperable, and valuable knowledge would remain in free-text form in the literature. Curation is multi-faceted and includes applying appropriate metadata, mapping to standard identifiers and extracting structured knowledge from the literature.

#### Use what already exists, collaborate with existing groups

The current metadata landscape is complex and varied as many groups attempt to solve the problem anew. There is no need to start fresh; contribute to existing groups and recommendations. Use and help develop best practices.

#### Create a robust network of groups working on metadata

The Metadata and Ontologies Working Group, a sub-group under the AgBioData consortium, is a good place to start. It will be difficult or impossible to find and collaborate with everyone working on metadata, but a network will help to encourage collaboration and to raise visibility of the metadata issue. This network will help newcomers understand who is doing what. The situation now is bewildering to someone trying to use metadata correctly. FAIRSharing, COPO and CyVerse are expected to be good partners in the task of disentangling overlapping standards, protocols and tools.

#### Improve database compliance

Each GGB database should require metadata for data sets they host. They should provide metadata in both human readable formats and machine readable. Metadata should be included with any web service API provided by a database. GGB databases need to help their users find and use appropriate metadata e.g. by embedding the BioSharing search widget in web pages.

#### Adopt a profile approach

Take what you need from existing standards and link to well-established repositories (e.g. BioSamples for sequencing data, genotypes, gene expression etc.) Two groups that are likely partners in this are FAIRsharing and CyVerse.

#### Encourage and enforce use of persistent identifiers for data sets

 Journals could require established Persistent Uniform Resource Locator (e.g. DOIs, ARKs, accessions from permanent repositories). Also archive historical identifiers (e.g. as synonyms) and link to persistent identifiers.

#### Improve researcher compliance

Create incentives for researchers. Work with funding agencies, journals, databases and general repositories to enforce the use of metadata standards. Work with data librarians to develop and present workshops and tutorials to train scientists via webinars.

#### Identify and use critical shared identifiers

It is necessary to link resources, for example, linking germplasm with genetic variants. In this example, it should be mandatory to have common identifiers in both the germplasm/stock centers and genomic resources. These could be gene IDs or genomic positions. Other examples are linking gene model IDs across organism and clade resources, as used in gene families; and common trait names within, and to the extent possible, across related species.

#### Collaborate when creating new metadata standards

Joint pilot projects should be initiated to assess requirements and establish best practices when new data types are anticipated and standard methods for data integration are not already available.

**Explore tools and processes that can ease the burden of metadata collection** for both data curators and researchers.

## GGB database platforms

### Overview

Tools that allow biologists to store, distribute and visualize biological data are essential for collaborative and effective science. This need was identified early on with the advent of whole-genome sequencing. Several tools and database schemas were developed to meet the needs of GGB databases, including AceDB in 1989 for the *Caenorhabditis elegans* genome (58), which was adopted by and modified for other projects; NCBI Entrez (59) released as an online resource in 1993; Ensembl (60) in 1999; the UCSC genome browser (61) in 2000; Gadfly for *Drosophila melanogaster* (62) in 2003; AtDB/Illustra in 2003, followed by TAIR (63) for *Arabidopsis thaliana*; and the Genomics Unified Schema, released in 2005 for *Plasmodium falciparum* (64). For germplasm, the Germplasm Resources Information Network (GRIN)-Global platform was initiated in 2009 (65), and has since been adopted internationally for other genebanks (www.grin-global.org/).

With low-cost next-generation sequencing technologies, generating genome assemblies is now possible for organisms with smaller research communities and fewer resources. In addition, technologies enabling high-throughput phenotyping can generate large data sets that would benefit from being linked to genomic and genetic data for the same individuals and species. In light of these technological advances, genome-centric databases tailored toward smaller communities, and across multiple taxa, have proliferated (66), and several models for storing and accessing data have become adopted.

Here, we focus on platforms for community databases centered around GGB information. We define a ‘platform’ as the database schema and software that serves biological information to researchers. GGB information is usually stored in a database schema, contained within a DBMS (e.g. a relational DBMS like MySQL, a triplestore system like RDF or other noSQL DBMS such as HDF5 and Neo4j) and accessible for outside use via an application that interfaces with the DBMS.

#### Review of available platforms

There are multiple platforms currently available for building GGB databases. Here we review several platforms: three that are well established and three newer platforms that show promise. The criteria for selecting these platforms are the following:

1. Open-source codebase and schema;
2. Useful documentation available for installation, management and APIs (if applicable);
3. Adoption of the platform by more than one group, demonstrating that other groups can successfully set up the platform;
4. User support available (e.g. via GitHub issue tracker);
5. The platform enables public data access (e.g. not just a local installation).

The three ‘well-established’ platforms met all criteria; the three ‘promising’ platforms met four out of five criteria. This list is not meant to be comprehensive. Table 5 outlines the data types that each platform supports and rates how well the data types are supported by each platform.

**Table 5:** Data types and their support by each GGB platform. The following table rates support for each data type using the following scale: (x) No Support, (i) Schema Support Only, (ii) Extension Module Support and (iii) Core Interface Support. Core interface support implies the core application supports this data by providing loaders and front–end visualization. Extension module support implies this functionality has been added by some groups using the application and is now available in a sharable format (extension module or detailed tutorial). Schema Support Only implies the database schema can store this type of data but as of yet, no loaders or front–end visualizations are available

		**Well-established, recommended platforms**			**Promising platforms**			
		**Tripal**	**InterMine**	**Germinate**	**GenomeHubs**/**Ensembl**	**SGN**	**T3**	**Reactome**
Data category	Data type	Chado & PostgreSQL	PostgreSQL	MySQL	MySQL	Chado & PostgreSQL	MySQL	GraphDb (neo4J)
**Genomic**	Assembly	3	3	x	3	3	2	x
	Gene annotation	3	3	x	3	2	2	x
	Gene–gene interactions	1	3	x	x	2	x	x
	Protein domains	1	3	x	3	2	x	x
	HTS Gene expression data	2	2	x	3	3	x	x
	Array-based gene expression data	1	2	x	x	x	x	x
	RNA-seq	2	2	x	3	2	x	x
**Diversity**	Genomic variation (copy #, translocations, SNPs)	1	2	x	3	3	3	x
	Genotypic data (alleles, polymorphisms)	2	3	3	3	3	3	x
	Phenotypic data	2	2	3	3	3	3	x
	QTL	3	2	x	x	3	2	x
	Mutants (fast neutron, transposon, Ethyl methanesulfonate (EMS))	x	x	x	x	3	x	x
	Phylogeny	3	2	x	3	2	x	x
	Comparative analyses	1	1	x	3	3	x	x
**Germplasm management & breeding**	Germplasm Stocks	3	3	x	x	3	x	x
	Germplasm (Varieties, Landraces etc.)	3	2	3	x	3	3	x
	Germplasm pedigrees	2	2	3	x	3	3	x
	Genetic maps	2	2	3	x	3	3	x
	Field trial Data	1	1	3	x	3	3	x
**Other**	Pathways	x	3	x	2	2	2	3
	Images	2	2	3	x	3	x	x
	Ontology	3	3	x	2	3	3	x
	Ontology-based annotations	3	3	x	3	3	3	x

**Well-established platforms: Tripal plus Chado**

Tripal (42, 67) is a freely available, open-source platform for building community-focused, biological data web portals (http://tripal.info; https://github.com/tripal/tripal; GNU General Public License v2+), and is currently in use by many plant and animal genome databases (http://tripal.info/sites_using_tripal). Tripal extends Drupal, a popular content management system, to provide a robust, modular platform with a powerful administrative user interface, extensive developer APIs and advanced website functionality. Biological data is stored in the Generic Model Organism Database (GMOD) Chado database schema (68) that provides flexible, ontology-driven storage for most biologically relevant data types. Paired with PostgreSQL, Tripal and Chado provide high data integrity while the Drupal Storage API provides the flexibility to use alternative storage solutions when necessary. Rather than providing a data warehouse, Tripal focuses on building full community web portals with integrated biological data storage.

Installation of Drupal and Tripal is extremely well documented and includes an automated portion guiding the administrator through the initial setup and basic configuration. This makes the basic setup of a Tripal site extremely easy. The process does, however, become more complicated once data loading and configuration of extensions begin due to the large number of options available. Another area that can be overwhelming is the extensive combined Drupal/Tripal Developer API that allows site developers to customize every aspect of a Tripal site. This API is a huge advantage insofar as it ensures that anyone can customize a given aspect or add a given feature; however, its size and complexity can overwhelm developers new to the platforms. Luckily, it is well documented with many tutorials, and Tripal has an active, helpful developer community (see http://tripal.info/support). However, it is important to keep in mind there will be a learning curve for even an experienced PHP developer.

The GMOD Chado schema is another area of complexity. In order to achieve flexibility, Chado is a normalized schema divided into modules by data type. Each module typically has one or more base tables with minimal fields, extendable by property tables, and a set of linker tables to tie data objects together. This allows Chado to support data types that did not exist during the design phase, but also results in the need for many table joins to extract the data for dissemination. Since queries with a large number of joins can be a performance issue, custom indexes and materialized views are often used; however, this results in data duplication and increases database size. Overall, Tripal’s many features, extensions and customizations paired with its active developer community make the investment to evaluate this platform well worth it.

**Well-established platforms: InterMine**

InterMine is an open-source data warehousing system that was originally developed for FlyMine (69, 70), and now has become widely used for other model organism databases (71–77). The InterMOD consortium, a collaboration that includes the development teams for InterMine and five model organism databases, has worked to provide a platform for cross-species analyses through FlyMine, MouseMine, RatMine, ZebrafishMine, YeastMine and WormMine (78). InterMine is increasingly being used for organisms important to agriculture, e.g. MedicMine (79) for Medicago truncatula, HymenopteraMine (80), BovineMine (81) and others.

Advantages of InterMine are the following: (i) it enables the integration and fast mining of large complex data sets; (ii) the database schema is customizable; (iii) data can be accessed via the InterMine web application and custom applications; (iv) the InterMine system includes a collection of parsers (28 parsers as of 2013) (82) to load data from typical data sources; and (v) InterMine includes an identifier resolver system to address issues that emerge due to changing identifiers for updated gene models. InterMine is not designed for incremental data entry or for data curation. Furthermore, the schema denormalization that enables high query performance can be a disadvantage for storing primary genome and annotation data because data integrity cannot be enforced when the database is denormalized. Therefore, it is not uncommon for a model organism database to employ both InterMine for data mining and a different schema, such as Chado, for storing primary genome and annotation data sets.

In deciding whether to use InterMine, one should consider the diversity of data available for a species and whether users would benefit from integrating the genome assembly and primary annotation with other data types. It may not be worthwhile to set up InterMine for a newly sequenced organism, unless a more established model organism can be leveraged via orthology. InterMine is suitable for integrating gene sets of non-model organisms with orthologs in model organisms and the additional information connected to the model organism genes, such as pathways and gene interactions.

The core of the InterMine platform is an object/relational mapping system, the ObjectStore, which is optimized for read-only database performance. It accepts queries from a client and generates SQL statements, which are optimized by a Query Optimizer and executed in the underlying database. The Query Optimizer enables high performance by automatic denormalization, i.e. the generation of pre-computed tables of connected data after all data is loaded. Data can be accessed through Representational State Transfer (RESTful) web services and the InterMine web application. Client library support is provided in Python, Perl, Java, Javascript and Ruby. InterMine relies on the SO as its core biological model.

The InterMine platform is well documented with tutorials (http://intermine.readthedocs.io/en/latest) for configuring and setting up an instance. The InterMine Registry (http://registry.intermine.org) improves visibility of existing instances of InterMine while the InterMine Blog (https://intermineorg.wordpress.com) updates the community on its development roadmap and future releases. Researchers can contact developers and the community at large via the GMOD InterMine mailing list or via Discord. The codebase for InterMine is available on GitHub (https://github.com/intermine/intermine), which also has an issue tracker for requesting features, raising issues and development-related discussions.

**Well-established platforms: Germinate**

Germinate is an open-source platform (BSD 2-Clause License) designed for storage and access of data related to genetic resource collections (https://ics.hutton.ac.uk/get-germinate/; 79, 83). The Germinate platform supports data types relevant to genetic resource management, including phenotypic, genetic, geographic, pedigree, field trial and ‘passport’ metadata. There are various methods for data display available, and the platform was designed to be extensible. The latest version is implemented in the Java-based Google Web Toolkit and MySQL. The platform is well documented (http://ics.hutton.ac.uk/svn/germinate3/trunk/documentation/germinate-documentation.pdf), with a Docker image available. The project does not have a public issue tracker, but comments and queries can be submitted to germinate@hutton.ac.uk.

The Germinate platform is tailored toward breeding collections, and therefore is not a good fit for a database that is more sequence-centric (e.g. genomes and their annotations). However, for breeding and genetic resource data, the platform is well documented with fairly fast performance for a variety of project sizes, and nice visualizations of a variety of data types. The platform is compliant with the Multi-Crop Passport Descriptors standard developed by the Food and Agriculture organization. If new data types that are not covered by the current schema implementation need to be stored, it should be possible to add new subschemata.

**Promising platforms: GenomeHubs from Ensembl**

Ensembl is a well-established system to analyse, store and access genomic data sets (84). Ensembl websites provide a consistent user interface to access genomic data. Examples include Gramene (85) and Ensembl Plants (86) for plants across taxonomic clades and Ensembl (39) for vertebrates. However, the challenge of installing the Ensembl system and loading data into the database has discouraged many GGB databases from adopting the Ensembl database schema and/or codebase. GenomeHubs was developed to ease adoption of Ensembl (87). GenomeHubs is open source, provides a containerized setup of the Ensembl database schema and webserver and offers scripts to load genomic data into the database. GenomeHubs provides detailed documentation on its website, and user support is available via GitHub (https://github.com/genomehubs/genomehubs/issues). While fairly new, GenomeHubs already represents a promising path to democratize access to the Ensembl system for most genome projects. Because the Ensembl schema is primarily sequence-centric, this platform may not be a good fit for breeding data.

**Promising platforms: Sol Genomics Network**

The Sol Genomics Network (SGN, (https://solgenomics.net) is a web portal for sequence and phenotype data, as well as a variety of analysis tools (88). This open-source platform (MIT license) is currently tailored toward plants in the family Solanaceae, and has been expanded to a comprehensive breeding management system emphasizing next-generation breeding technologies, such as genomic selection. GGB databases that use this platform include Cassava (https://cassavabase.org), yam (https://yambase.org), sweet potato (https://sweetpotatobase.org) and banana (https://musabase.org). Another implementation of the SGN platform is https://citrusgreening.org, which focuses on interactions of host, vector and pathogen. SGN is recommended especially for communities that produce both sequence and phenotypic data for breeding programs. A standalone installation is available on GitHub (https://github.com/solgenomics/solbase). SGN staff is happy to provide technical support for prospective adopters.

**Promising platforms: T3**

T3 (https://triticeaetoolbox.org) is a database and website designed to enable plant breeders and researchers to combine, visualize and query phenotype and genotype data across contributing plant breeding programs (89). Data is loaded using Microsoft Excel or text file data templates. There are data templates for importing germplasm lines, trial means, plot-level results, field layout and canopy spectral reflectance. Analysis tools integrated into the website include GWAS, Genomic Prediction, Selection Index and statistical reports. T3 enables users to define data sets for download in formats compatible with external tools such as TASSEL, Flapjack and R. T3 is freely available at https://github.com/TriticeaeToolbox/T3, and installation instructions are posted at https://triticeaetoolbox.org/wheat/docs/INSTALL.html. A typical installation includes a sandbox server used for testing imports and a production server that is used for public access.

**Promising platforms: Reactome**

Reactome (https://reactome.org) is an open-source, open access, intuitive platform for the visualization, interpretation and analysis of pathways (90). Reactome supports manual curation of reactions and pathways, using appropriate ontologies, and cross-references to other public resources, including NCBI, Ensembl, UniProt, ChEBI and PubMed. The Reactome platform was initially developed for human pathways and was later extended to pathways for several other organisms, including cattle (*Bos taurus)*, chicken *(Gallus gallus),* dog *(Canis familiaris)* and swine *(Sus scrofa).* Software and instructions for installation of the Reactome database, website and data entry tools are available for independent pathway curation (https://github.com/reactome). Recent developments at Reactome have led to adoption of a graph database (Neo4j) and a new ContentService (REST API) to build the query system (90). The Gramene database adopted the Reactome data model and platform to create the Plant Reactome portal (http://plants.reactome.org), which currently hosts a collection of plant pathways from 75 species (13).

### Challenges and opportunities

**Platform choice.** Often, GGB databases are focused on information pertaining to a single species or a single clade. While general-purpose databases and repositories do exist and should be used, in particular for sequence data, community databases fulfill needs specific to non-model organisms, often with a unique set of data types. These needs can include non-genetic or genomic data types (e.g. phenotypic data); expert or community curation of gene models; pathway databases; metadata and ontologies tailored toward a particular community or research focus; detailed queries of the data; community interactions regarding the data; and means for finding all data within a taxonomic group. Since GGB databases are built to meet specific needs of their community, the requirements for infrastructure and software can vary widely, even though multiple communities would benefit from shared platforms. Not only do the requirements for infrastructure and software vary among GGB databases, GGB databases also need to continuously update their platforms to accommodate new types of data generated from new technologies.

**Platform sustainability.** It is common that GGB databases need to be built in a short time, with no prospect of continued funding. Building and maintaining a platform for GGB databases is a challenging task due to the complexity and the volume of the data, requiring extensive collaboration between experienced programmers, database/system administrators and researchers, especially when the GGB database is built from scratch. Sustaining a GGB platform over time is therefore a challenge.

**Platform interoperability.** GGB databases use a diversity of database schemas, many employing existing platforms, custom-built solutions or a combination of both. The diversity of platforms used can pose challenges for data exchange and interoperability among databases.

### Recommendations

#### Recommendations for platform choice

**Things to consider before choosing a platform.**

First, the developers need to have a good understanding of the data that will be stored in their community resources, and have a plan for how the data will be discovered and transferred. This includes understanding the data types and their associated metadata, as well as the relationship between different types of data. The developers also need to know whether the data are standardized by the producers and what additional standards will need to be applied to maintain full data integrity. This should encompass versioning, naming, metadata standards and ontologies. (Recommendations for all of these are covered in significant depth elsewhere in this document.) Additionally, the developers will need to plan ahead for appropriate data capacity, considering both the total size of the target data set to start and how fast the data is expected to grow. This can be difficult to accurately predict—the existing data in flat files may expand significantly when moved into a relational database, and advances in instrumentation that lead to huge increases in data production are common. We recommend looking at past trends, talking to leading minds in the field and running tests of storage solutions to make these decisions, then leaving significant extra capacity on top of the best predictions.

Beyond the data, it is critical to develop a clear picture of researchers and their needs. Researchers may have a variety of roles, each of which must be considered separately. In each of these roles, researchers may have different needs for downloading, visualizing, searching, editing, analyzing or otherwise interacting with each data type. Further, these roles may span different expertise levels, from new users and students to power users who run complex and sophisticated analyses. To develop a clear understanding of the researchers and the tools needed, use cases are a powerful tool and are highly recommended.

Next, the local resources and the development environment need to be considered. How much development capacity is available? The number of available developers, including local IT professionals, their expertise, along with the timeframe for development, informs the scope of what can be successfully delivered. If funding is short term, a long-term plan for maintenance and sustainability may need to be developed. In this case, an open source solution may be particularly attractive, to enable later development and maintenance by different developers.

Hosting services vs. hardware administration and backups, while outside the scope of this document, are also critical considerations. Further, a thorough understanding of local policies is critical for initial decision making. The local institution, the funding agency and the data itself may have licences, ownership and other conditions that need to be thoroughly understood.

**Systematically choose from available platforms.** Table 5 represents a guide to selecting from the available platforms listed above. This table objectively compares the data support for these platforms. The intent of this table is to allow readers to prioritize the platforms that have at least schema support for specific data types.

Consider the following example: a research community has decided to fund a community resource to house a recent genome assembly, including annotation, as well as a collection of genotypic and phenotypic data for a core set of germplasm. Their highest priority is a platform that will handle genome assemblies, gene annotation and genotypic and phenotypic data. The genotypic and phenotypic data includes a number of bi-parental crosses and, as such, it is likely that the community will also want to store genetic maps and QTL data in the future. According to Table 5, both Tripal and InterMine support these data types, making these platforms worth considering further.

In addition to supporting different data types, platforms come with different built-in feature. Table 6 compares features provided by the reviewed platforms. Rather than considering all the features and choosing the most feature-rich platform, it is recommended to focus on the features important to the platform’s intended users. Considering the previous example, this may have the following prioritized feature list: (i) community-building tools; (ii) easy search functionality; (iii) genome browser and BLAST; (iv) GWAS visualizations such as Manhattan plots; and (v) advanced querying for a subset of users. Table 6 shows that both Tripal and InterMine provide easy search functionality, genome browser and BLAST integration. However, Tripal excels at community-building tools, whereas InterMine excels at advanced querying. This example highlights that the best solution might be a collection of platforms depending on how diverse the users’ needs are. Finally, consider the last feature, GWAS visualizations, which is not included in Table 6. Remember that if a given platform does not include an important feature, it is always worth contacting the developer. They are often happy to receive feedback and may even be in the process of implementing new features of interest. Furthermore, keep in mind that it will require less development to add a feature to an existing platform than it will be to develop a new platform.

**Table 6:** Feature sets supported by each GGB platform. The following table rates support for a comprehensive list of features with the following scale: (x) no support, (⭑) extension module support and (✔) supported by core. You should highlight which features are most important to your user group to ensure that you choose the platform best suited to your needs. Core interface support implies the core application supports this data by providing loaders and front–end visualization. Extension module support implies this functionality has been added by some groups using the application and is now available in a sharable format (extension module or detailed tutorial)

		**Well-established, recommended platforms**			**Promising platforms**			
		Tripal	InterMine	Germinate	GenomeHubs/Ensembl	SGN	T3	Reactome
Feature category	Feature type	Chado & PostgreSQL	PostgreSQL	MySQL	MySQL	Chado & PostgreSQL	MySQL	GraphDb (neo4J)
**Query**	Simple full site keyword search	2	2	2	2	2	2	2
	Question-based searches (set up by administrator)	2	2	2	x	x	2	x
	Simple advanced searches (multiple fixed filter criteria)	2	2	2	x	2	2	2
	Query-builder advanced search	x	2	2	x	2	2	2
	List support	1	2	2	x	2	2	x
	Genomic region search/overlap queries	1	2	1	2	1	1	x
**Browsing**	Basic browsing	2	2	2	2	2	2	2
	Data information pages (e.g. gene pages)	2	2	2	2	2	2	2
	Data type-specific summaries	1	2	2	2	2	2	2
**Community**	Forum	1	x	1	x	2	1	x
	Conference pages	2	1	1	x	x	x	x
	Community news	2	2	2	x	2	2	x
	Community curation	2	x	x	x	2	x	x
	Community information pages	2	1	2	x	2	x	x
**Data sharing**	Web services	2	2	2	2	2	2	2
	Query across sites	2	2	1	x	2	1	2
	Reference sister sites	2	2	2	2	2	2	2
**Other**	Genome browser integration	1	2	1	2	2	1	x
	BLAST integration	1	1	1	2	2	1	x
	Access control (login system)	2	2	2	2	2	2	x
	Data analysis tools	1	1	2	2	2	2	2

#### Recommendations for platform sustainability

**Use open-source tools if possible** (91). If open source tools do not perfectly meet the need, we recommend reaching out to and collaborating with tool developers to see how database needs can be integrated with existing efforts. Federal funding agencies such as the NSF and USDA have encouraged developers to build on each other’s work and thus multiply the scope and impact of their efforts through shared code. If you develop your own tools, publish your code and documentation in a public hosting service such as GitHub, BitBucket or SourceForge.

**Have a plan for long-term management.** Things to consider are how the resource will be maintained when no funding is available, what open-source tools would make it easier for long-term maintenance with little funding and what hardware resources are needed in the future. For long-term maintenance, databases built on shared platforms could be (more) easily integrated and potentially maintained at a lower cost.

#### Recommendations for platform interoperability

**Employ ontologies and CVs and use them to annotate as much data as possible.** Where community-derived CVs are not available, we recommend reaching out to others before creating custom vocabularies. When custom vocabularies are needed, it is best to design them with the intent to either submit them for inclusion in an existing vocabulary or make them publicly available.

**Plan for web services.** We recommend using web service APIs that are already widely adopted, for example the Breeding API (BrAPI; http://www.brapi.org; https://github.com/plantbreeding/API), and to work with other groups who may want to consume data from the site being built.

**Make your database discoverable by indexing and providing a search engine.** This will expose your data through a standard interface and allows creation of a federated database system. Two methods of implementing this are Apache Solr: https://lucene.apache.org/solr and BioMart: http://www.biomart.org (92).

**Make your data connected using best practices for exposing, sharing and using Uniform Resource Identifiers and RDF.** A list of resources for these methods is at http://linkeddata.org and https://www.w3.org/DesignIssues/LinkedData.html (93).

## Programmatic access to data

### Overview

A key component of FAIR data principles (4) is that data can be found, read and interpreted using computers. APIs and other mechanisms for providing machine-readable (i.e. programmatically accessible) data allow researchers to discover data, facilitate the movement of data among different databases and analysis platforms and when coupled with good practices in curation, ontologies and metadata are fundamental to building a web of interconnected data covering the full scope of agricultural research. Without programmatic access to data, the goals laid out in the introduction to this paper cannot be reached because it is simply not possible to store all data in one place, nor is it feasible to work across a distributed environment without computerized support. After a brief description of the current state of data access technology across GGB databases and other online resources, we more fully describe the need for programmatic data access (also referred to as ‘machine access’) under Challenges and Opportunities and end with recommendations for best practices.

Sharing among AgBioData databases is already widespread, either through programmatic access or other means. The results of the AgBioData survey of its members indicate that GGB databases and resources vary in how they acquire and serve their data, particularly to other databases. All but 3 out of 32 GGB databases share data with other databases, and all but two have imported data from other database. Some make use of platforms, such as InterMine (69, 70), Ensembl (84) and Tripal (42), to provide programmatic access to data that is standard within, but not across the different options. Other databases develop their own programmatic access or use methods such as file transfer protocol (FTP: a standard network protocol used for the transfer of files on a computer network). Finally, some databases provide no programmatic access to data. A number of infrastructure projects already exist that support AgBioData data access needs, most of which have been adopted to some degree by different GGB platforms (Supplementary Material 5).

A more recent approach to facilitate data search, access and exchange is to define a common API that is supported by multiple database platforms. An example of this is BrAPI (https://brapi.org), which defines querying methods and data exchange formats without requiring any specific database implementation. Each database is free to choose an existing implementation (if compatible) or to develop its own. However, BrAPI’s utility is restricted to specific types of data. Alternatively, the Agave API (94) provides a set of services that can be used to access, analyse and manage any type of data from registered systems, but is not customized to work with GGB databases.

### Challenges and opportunities

Aside from primary repositories like GenBank, model organism and specialty databases remain the primary means of serving data to researchers, particularly for curated or otherwise processed data. These databases represent different community interests, funding sources and data types. They have grown in an *ad hoc* fashion and distribute data in multiple formats, which are often unique to each database and are may be without programmatic access. Below, we lay out some of the challenges and opportunities in programmatic data access faced by GGB researchers using the current landscape of databases. Exploration of these use cases yielded a set of common data access requirements under five different themes, summarized in Table 7.

**Table 7:** Requirements for programmatic access to data in the genetics, genomics and breeding community

Theme	Requirements
Discovery	1. Web services for discovery of available resources 2. A way to search data across many resources 3. Good API documentation describing programmatic access
Data and metadata	4. Common file formats 5. Common classification systems (e.g. consistent use of same gene families and ontology terms) 6. Ability to access and combine data, retaining provenance and metadata such as species of origin that will be of interest in the aggregated context 7. Machine readable metadata
Authentication	8. Shared authentication protocols 9. Authentication through use of keys
Data exchange/ transfer	10. Web services to extract data from any compatible database 11. Services to deliver data to another database or end users 12. Easy data transfer from NCBI (currently requires installation of a specialized tool) 13. Data provenance tracking 14. Data usage tracking via web services 15. Data management support for distributed data
Remote analyses	16. Data staging (temporary storage) for analysis platforms 17. Access to computing resources 18. Request status polling (mechanisms to automatically report the status of an operation)

**Comparative genomics across species requires access to distributed data.** Large comparative genomic portals exist (e.g. Phytozome, Ensembl, PLAZA) but have limitations in their utility for specialized communities, such as not incorporating data from minor crop species or crop wild relatives or rarely handling multiple genomes for the same species. Distributed and independent genome projects produce assemblies and annotations that can be beneficial to research on related species, if researchers can discover them. However, even a multi-species database that manages gene families may not contain all gene data of interest to the communities it serves. Services that assign new data, supplied by researchers or by other sites, to gene family memberships can help with discovery across databases by putting new sequence data into an evolutionary context, but then the data must be discoverable broadly.

**Different genome assembly versions exist.** In GWAS studies, a scientist may be looking for candidate genes near SNPs with significant association to a trait of interest, but find that annotation data in a multi-species database comes from a different assembly version than was used in the study. Possible solutions include (i) the database provides access to annotations on the assembly version used in the study, (ii) the scientist uses a batch coordinate conversion (e.g. LiftOver; https://genome.sph.umich.edu/wiki/LiftOver) to convert coordinates to the new assembly or (iii) the scientist repeats variant calling and association analysis using the new assembly. The first approach requires the database to offer programmatic access in the form of web services to many versions of assemblies and annotations. The second and third approaches require access to significant compute power, thus requiring databases to invest in computational resources (for solution 2) or to provide access to cloud compute resources with some level of authentication and scalable data transfer (for solution 3).

**Integrating data and computational analysis.** Applications that can operate where the data exists, to support comparative access for pre-publication and privately maintained genomes, can reduce the need to move large data sets among locations. For example, a group might generate a draft assembly of an accession with a novel phenotype that they have mapped to a certain genomic region. They may then wish to compare the scaffolds that contain the region of interest to a reference assembly for a different accession or for a related species, to find candidate genes that may be novel to their accession. Existing services such as CyVerse (95) can be used to analyse data from many sources. Being able to do the comparison where the different genomes are located would save moving and duplicating large genome files, but requires considerable investment in distributed computation.

Another solution is for GGB databases to host a local Galaxy (96) instance connected to a Tripal database (42) with public and private data sets. This is effective if a researcher with phenotypic, genotypic and environmental data (e.g. for GWAS analysis) needs a place to house the data both before and after publication, but is not an expert in genomic analyses or data management. Analysis pipelines tailored to the needs of a particular community, hosted through that community’s database, allow researchers to upload, search and visualize private data and public data, select these data and parameterize an association mapping workflow and execute that workflow locally. In order to execute the analysis remotely, data will need to move efficiently from the database to a remote analysis platform.

**Data Discovery across platforms.** Scientists often want to discover all that they can about a particular entity (gene, gene model, phenotype, species), but the data are distributed across multiple resources, many of which may be unfamiliar. Each data element on its own is not large, but the total space to be searched is. A hypothetical workflow is as follows: a researcher who works on one species comes to a participating database with a sequence of interest, wanting to find out what biological functions their sequence might be involved in. The researcher identifies homologous sequences in the new database by running BLAST. The database converts the BLAST results to an exchangeable token and queries other databases for information about orthologs. The product of these requests could be as simple as a gene name/symbol and a URL to point the user to the data display at the external database, or could also include provenance and database information for attribution, sequence, publications and many other types of information. For data discovery to work, databases with relevant data and compatible APIs must be discoverable and well documented, and a method should be in place to track usage across different services.

### Recommendations

Below we present a specific list of recommendations for programmatic data. Numbers in parentheses after each recommendation correspond to the needs outlined in Table 7. Although the examples discussed above focus on large and/or complex data sets accessed via APIs, some improvements can also apply to databases that are accessed primarily through a web interface.

**Move to a federated model of data exchange.** Many of the current challenges with data discovery and exchange could be addressed with a ‘federated’ model whereby a group of databases has voluntarily agreed to open their data for programmatic access to each other through an API. While the federation does not have the power to compel participation, it does give data providers another source of traffic to their sites and data. At a time when database usage metrics are used by agencies for consideration of continued funding, this is an attractive inducement. Federation also addresses the API standardization problem. Also, the federation can then serve as a common point of advertisement for each participant’s services, which in turn could encourage other providers to participate in the federation. (Table 7; requirements 1, 2, 6, 7, 8, 9, 10, 11, 12, 14 and 18)

**Use standard transfer protocols, but use modern, efficient ones.** If transfers must occur via the web, databases should use https instead of http. Many databases serve their data primarily as complete files via FTP, which means these files are accessible, but not findable or interoperable. While FTP sites can be accessed programmatically, we recommend that databases move toward federated data exchange. Furthermore, FTP is not the most efficient transfer method and may not work well for larger data sets (e.g. annotated genomes). In contrast, http(s) and other methods such as The Integrated Rule-Oriented Data System (https://irods.org/) allow for a variety of optimizations such as byte-range requests to facilitate indexed access. (Table 7; requirements 1, 3 and 10)

**Provide a direct connection to data.** It is often not feasible for researchers to download data to their own workstations or institution servers for analysis. High-throughput data (genomics and phenomics), because of their size, need a direct connection to analytical resources. CyVerse (95) and Tripal/Galaxy (https://www.drupal.org/project/tripal_galaxy) are two web-based solutions for transfer and analysis of large data sets. (Table 7; requirements 16, 17 and 18)

**Minimize authentication hurdles to data access.** Authentication is the process of verifying the identity of a user attempting to log in to a database and insuring that the user is authorized to do so. While authentication is useful to databases because it allows usage tracking, it severely limits programmatic access to data. Therefore, for public data we recommend Genetic and Genomic Database (GDBs) do not require authentication. If a database does require authentication to access data, GGB databases should move toward a single authentication service (or a small suite of authentication services), and the community should pick one or a few (e.g. OAuth or systems that use OAuth such as CyVerse, Github, ORCiD, Google). API specs must accept authentication details and be well documented. (Table 7; requirements 3, 8 and 9)

**Store data in a manner that is consistent with community standards and adopt appropriate ontologies to allow cross-query and integration across platforms.** A specific focus on applying and maintaining appropriate metadata is critical. As experimental methods change, the information associated with raw data also changes. (Table 7; requirements 4, 5, 6, 7, 13 and 15)

**Select one or more database schemas and APIs that are well supported by the development community.** Community-supported generic relational schemas (e.g. Chado) should be used where possible to reduce duplication of effort. Such schemas capture the majority of use cases related to common genetic and genomic data types. They also shorten the time necessary to optimize data storage and exchange. Current efforts in the community are focused on APIs that support the best data storage mechanism for the data (optimized relational schemas, NoSQL solutions and/or data warehousing) to facilitate access. In these cases, web services are developed to expose and share this data. (Table 7; requirements 4, 5, 6 and 7)

**Provide explicit licensing information that specify download and reuse policies.** No license is legally the same as applying the most restrictive license possible. Therefore, individuals and databases that want to make data available for reuse should formally apply the least restrictive license that makes sense for their data. The most frequently used licenses for data come from the Open Data Commons (https://opendatacommons.org/) and the Creative Common (https://creativecommons.org/share-your-work/licensing-types-examples/). GGB databases should have practices and policies that encompass the following:

- Exporting license and conditions (metadata) with the data,
- Requiring data submitters to agree to licensing and reuse policies,
- Recommending open access licenses or placing data in the public domain,
- Ensuring that curators identify licenses for data sets that they gather,
- Helping data submitters communicate with their institution about whether they can offer specific licenses given intellectual property rules (Table 7; requirements 6, 13 and 14).

**Work with your IT department to access high speed Internet.** This often involves moving your database outside a firewall. If appropriate, collaborate with other databases to create transfer links on Advanced Layer 2 Service (AL2S) to speed transfer. AL2S, as part of Internet 2, provides research and education organizations with access to effective and efficient wide area 100 gigabit Ethernet technology (https://www.internet2.edu/products-services/advanced-networking/layer-2-services/#service-overview). (Not specific to any individual requirement, but necessary for success of most of them.)

**Capture usage metrics, including requests through APIs.** Some methods for tracking site usage (e.g. Google analytics) are driven primarily by mechanisms involving webpage loads and require additional tooling to be able to handle tracking of web services calls, so development of both useful metrics and methods of capturing them are needed.

**Ensure that continuous updates and efforts toward integrating with existing web services and ontologies are followed.** This requires continuous development and interaction with the community of developers working on these APIs. (Not specific to any individual requirement, but necessary for success of most of them.)

## Communication

### Overview

Improved communication between people working at GGB database is critical to successfully implementing the recommendations made in this paper. In addition, improved communication with people in the research community, funding agencies and journals is critical to the maintenance and reuse of research data.

There are **four main types of communication** that personnel at GGB databases should consider: (i) communication across GGB databases, (ii) communication with experimental data generators and researchers, (iii) communication with funding agencies and (iv) communication with journals. Here, we consider challenges and recommendations for each communication type in turn.

### Challenges and opportunities

**Communication with other GGB databases.** Similarity in the challenges that each GGB database faces (described in the previous sections of this document) means that we have to view each other as collaborators rather than competitors. However, until now there have been few mechanisms and incentives to foster such collaboration.

**Communication with researchers.** Communication with researchers must occur in both directions. GGB databases need to effectively communicate their scope and requirements to users. For example, many researchers, however well intentioned, simply do not know best practices for data management and/or how to find long-term storage for their data. GGB databases need to be more proactive in communicating with researchers to learn the researchers’ needs and to prepare for emerging research. GGB databases can also participate in communication between researchers and data librarians to help researcher craft realistic Data Management Plans. It may not be clear to the researcher how to reach out to the GGB database staff to provide feedback or that such feedback is welcome. Many GGB databases often do not have the funds to accomplish all of this on their own.

**Communication with federal funding agencies.** Federal funding agencies supporting GGB databases include USDA-ARS, USDA, NSF and NIH. While it is clear that these agencies value the role of GGB databases, there are several areas where increased communication with funding agencies would better enable our mission of facilitating scientific discovery and application in agriculture. Challenges occur on two levels: (i) how should proper data submission at GGB databases be encouraged and measured; and (ii) how can GGB databases communicate their challenges and the importance of their work to the funding agencies, in order to be considered for competitive grants.

**Communication with publishers.** Publishers clearly have an important role to play in making data publicly available and some journals have formed a joint data archiving policy (97). Most publishers have DNA (genome or gene) data submission policies. In contrast, some forms of research data such as maps, QTL and markers cannot be submitted to primary repositories (such as the INSDC or NCBI), and few peer-reviewed journals require submission of this data to community databases ahead of publication. Even though journals have data submission policies, the majority only ‘strongly encourage’ data submission. Furthermore, some managing editors do not enforce the journals data policies. Additionally, many reviewers do not verify that the data has been submitted to the database if indicated in the manuscript. Furthermore, metadata required in primary repositories is often insufficient for appropriate reuse within custom databases.

### Recommendations

#### Recommendations for communication among GGB databases

Frequent communication among GGB databases is the primary way to ensure successful collaboration (98). We recommend the following guidelines to improve communication among GGB databases:

**Join AgBioData.** Individuals working at GGB Databases can join here: https://www.agbiodata.org. Members of the email list will receive monthly newsletters about upcoming AgBioData conference calls, meetings or events and interesting publications. Also, each member GGB database is listed on the website.

**Attend the monthly AgBioData calls.** Each member GGB should have at least one representative on the calls. Each database can actively participate in the selection of topics and speakers. In addition to providing talks relevant to GGB databases, the calls also provide a good forum for discussion. Presentations are recorded and posted at AgBioData.org and YouTube, however, members are encouraged attend the conference calls to participate in discussions.

**Share information and collaborate.** This includes, but is not limited to the following: best practices; data standards and standard operation protocols; data (to reduce redundancy); code and code development; and strategic plans.

**Create an AgBioData Advisory Board** to represent the databases at a higher level. Include federal, competitive programs, industry and researchers and have the AgBioData Steering Committee formally report through yearly reports on activities and progress toward improved communication, collaboration and funding acquisition.

#### Recommendations for communication with researchers

**Mechanisms for outreach.** There are several mechanisms for outreach to researchers. The most common form of outreach is meeting and conference attendance. With a large number of researchers at meeting and conferences GGB databases can use these opportunities for workshops, presentations or a database booth. GGB database brochures can be handed out during the meeting and conferences. However, there are a number of researchers that are unable to attend meeting and conferences so it is important that GGB database also use other forms of outreach. These include newsletters, mailing lists, blog posts and social media (i.e. Facebook and Twitter) to inform researchers about new tools or data, webinars, workshops and videos. These forms of outreach can be used together to reach a broader audience. Using social media during conferences and meetings with the appropriate hashtag can send information about new tools and data to researchers who cannot attend the conference. A prime example of this is the Plant and Animal Genome Conference, which has a strong social media presence.

**Make it obvious who is doing the work.** Many online resources and databases do not mention the people on their teams and only provide an anonymous contact form. Individuals working on a resources or database should be named on the website. Being anonymous creates a barrier to communication, and if contact/feedback forms don't generate a response, there is no further recourse for the researcher to get help. Providing individual staff contact information and even photographs makes it easier for researchers to target questions to the appropriate person. Photos can enable researchers to find curators at meetings, and in general encourage communication by putting, literally, a human face on the GGB resources. Building in dedicated time at workshops for a ‘meet the team’ event, well advertized in advance to the research community, is also recommended to increase engagement opportunities.

**Respond quickly to feedback**. Every GGB database should have a feedback form. The feedback should be replied to within a reasonable period of time, say 24 hours during business days, even if you simply acknowledge the receipt of the feedback and provide a date by which the issue can be resolved. Everyone wants to be heard too!

**Provide tutorials**. Many GGB databases provide tutorials and FAQs. We propose having a central Tutorial Section at AgBioData.org, where tutorials relevant to all GGB Databases are available. This could include tutorials on file formats, or the best repositories for specific data types.

**Communicate on data management.** GGB databases should work with librarians to develop data management training courses. In addition, online data management modules can be developed to be used in graduate education, perhaps offering some type of certification for successful completion, including long-term assessment of training effectiveness.

**Set up an advisory panel.** Implementing advisory panels comprised of active, elected representatives of the stakeholder community, with clear mechanisms for reporting activities and plans of work can allow for direct, constructive feedback from database stakeholders.

#### Recommendations for communication with funding agencies

**Form an interagency GGB Database Advisory Board.** The board will meet regularly to discuss and document challenges and opportunities for GGB databases, data submission, data availability, data curation, data sharing and sustainability.

**Collaborate on data management guidelines.** GGB databases and funding agencies should discuss ways to develop more comprehensive and rigorous data management plans and methods to verify proper data submission to representative databases.

**Engage in joint social media activities** to deliver effective impact statements to stakeholders, industry and lawmakers.

**Identify or create funding opportunities to support GGB database activities**. Develop a clear understanding of the needs that both data providers and GGB databases face for effective data management and reuse, and highlight or implement funding mechanisms to support these needs.

#### Recommendations for communication with publishers

**Enforcement and adherence of data policies.** Data policies are only as good as the enforcement and adherence of those policies. The GGB database community and others in the research community need to remind publishers, managing editors and authors of the importance of public data. For publishers that do not have clear data availability policies, AgBioData and cohorts can strongly encourage the publishers to establish clear data availability policies. For the journals that do have data availability policies, the managing editors and reviewers must help enforce the data availability policies. If an author fails to follow the journals data availability policies the reviewer is within his/her right to reject the manuscript. Data curators who encounter a paper lacking publicly accessible data without obvious reason (e.g. proprietary funding) should notify the journal editors. In addition, as a collective, AgBioData will negotiate with the publishers to change public data submission from ‘strongly encouraged’ to ‘required’.

**Inter-agency sponsored workshop for journal publishers and editors.** Overcoming the challenge of reliable data submission will require communication among representatives from the appropriate journals, GGB databases and funding agencies to establish guidelines and an easy-to-submit and police system for researchers and the journals/funding agencies and databases. This would likely be best initiated through an inter-agency sponsored workshop, followed up by regular meetings and assessment of effectiveness. Such a workshop could also develop ways to ensure journal publishers and editors are aware of all relevant GGB databases so they can direct authors of each accepted paper to the proper repository, nomenclature clearing house etc. Providing access to centralized cyberinfrastructure where databases, journals and funding agencies could sign off on successful data submission for projects would help make this process easier for all parties and ensure accountability.

## Sustainability

### Overview

The GGB databases that currently comprise the AgBioData Consortium were created to serve the needs of researchers for access to curated and integrated data and analysis/visualization tools to aid scientific discovery, translation and application. The funding for these databases, however, is limited and not stable. Maintaining these resources in the longer term so that invaluable data are kept up-to-date and do not get lost is a major issue facing almost all AgBioData databases, their researcher communities and funding agencies.

### Challenges

AgBioData databases are supported through a variety of sources. Generally these fall into one of four categories: (i) primarily supported through line-item government funding, such as the USDA-ARS databases MaizeGDB, SoyBase, GrainGenes, Legume Information System and GRIN; (ii) primarily supported through competitive federal grants, such as TreeGenes, Hardwood Genomics, Gramene, Planteome, Solanaceae Genomics Network and Araport; (iii) supported through a combination of competitive federal grants, commissions and industry, such as the Genome Database for Rosaceae, AgBase, PeanutBase, AnimalQTLdb and CottonGen; and (iv) supported primarily through a user subscription model, such as TAIR.

With long-term government funding, the USDA-ARS databases enjoy the most stable financial support of the AgBioData databases. They typically represent high-value commodity crops serving a large research and industry community. While the level of support provided by USDA-ARS generally allows for continuation of base activities and curation, it typically does not provide resources for technical innovation or more resource-efficient systems to be implemented. For these, funding through competitive grants is increasingly necessary, as in the case of the NSF-funded Legume Federation award. At the other extreme lies TAIR, which after a phased withdrawal of support by NSF, successfully implemented a subscription-type funding model under a not-for-profit organizational structure (99). As the model plant for functional genomics, TAIR also has a large user community making this funding option more feasible to implement than for the databases represented in categories 2 and 3.

Many of the AgBioData databases have reported willingness of the scientific stakeholders to budget some funds in their grants to support data deposit and access to their community databases, similar in how they budget for peer-reviewed, open access publications costs. Unfortunately, most of the databases do not have organizational structures or processes that would allow them to accept these funds.

### Recommendations

While the purpose of this white paper is not to specifically recommend solutions to increase or stabilize funding of AgBioData databases, it should serve to start a meaningful and informed dialog with funding sources and research communities on sustainability of these resources.

**All the AgBioData databases should develop sustainability plans** through detailed strategic planning that actively includes stakeholders and funding agencies. These plans should detail and communicate the value proposition of their resource, construct budgets that plan for both subsistence-type operational costs and growth-type activities and even include exit plan strategies for the databases, should funding cease. The release of the ‘Process Guide for Data Repository Sustainability’ document by the Ecological Society of America following the NSF-sponsored Data Repository Sustainability Workshop held January 2018 should serve as a useful resource to develop these sustainability plans.

**Federal funding should be provided to support an overall review of AgBioData databases sustainability options**. This could be done by an organization that has already successfully gone through this process, such as Phoenix Bioinformatics for TAIR. One option might be to form a new not-for-profit organization, possibly through a partnership with Phoenix Bioinformatics, to create a process where researchers who budget support for data deposit and/or data access in their successful proposals can easily transfer these funds to their community databases through the not-for-profit. This would remove some of the current obstacles to accepting support from scientists that the databases face and provide a steady flow of funds once it become standard practice by scientists to include these in grants.

**Funding agencies should require a sustainability plan in proposals for new biological online resources.** Whatever series of options/solutions are developed to address sustainability of funds, it is clear that implementing these as a consortium is much more powerful and impactful. Funding more AgBioData workshops to further this dialogue will be critical to deriving solutions to the GGB database sustainability challenge.

## Conclusions

To enable all GGB databases to help their stakeholders utilize the increasingly complex biological data within and across the databases, we need to work together to adopt a common set of metadata, ontologies and communication practices; make it easy to share data; share curation practices; use common software platforms and components where reasonable; and provide solutions for long-term funding. The AgBioData consortium believes that the recommendations laid out in this white paper and continued communication between the people that power the many different GGB databases will allow us all to move forward in the right direction. Regular communication and transparency is the key for all these endeavors. While recognizing that it may be difficult for databases to comply with all of the recommendations outlined in this paper, a slow and steady push toward meeting the recommendations should be encouraged. This will not only make the work of biocurators more productive, it will improve the experience for researchers.

For all agriculturally relevant GGB database personnel, we recommend joining the AgBioData consortium, attending the monthly conference call seminars, joining a working group and working with your counterparts in other databases listed at agbiodata.org. A little time invested now in working together and applying common standards can save a lot of time later and prevent duplicated efforts in the development of tools and resources.

While the next step for AgBioData members is to work toward compliance with the recommendations outlined in this paper, we also realize that working closely with researchers, journals and funders is critical. Just as no one author of this paper was aware of all the resources mentioned herein, the situation can be even more confusing for scientists who primarily work at the bench or in the field, especially those not working in genomics. Training of both data and bench scientists and streamlining data management practices will get us closer to the goal of easy access to all data. We are all on the same page; the struggle is how to write that page!

## Supplementary Material

Supplementary Data

## Notes

**Database URL:** https://www.agbiodata.org
