# Supplementary material for: AgBioData consortium recommendations for sustainable genomics and genetics databases for agriculture
Source: Database (Oxford). 2018 Sep 18;2018:bay088. doi: 10.1093/database/bay088 (PMC6146126; doi:10.1093/database/bay088)
Supplement: Supplementary Data [file bay088_supp.zip › Supplementary Material1 through 4 resubmission.docx]

**Supplementary material 1: Survey results on ontology use in GGB databases**

To identify current status and challenges in ontology use, an online survey was offered to AgBioData members. Twenty-nine responses were received, twenty-five from AgBioData member GGB databases and four from non-member GGB databases. Some respondents represented multiple databases. The results of the survey demonstrated that the most common use of ontologies in curation was to associate sequence data with GO [[1]](https://paperpile.com/c/yG9wfR/OAbiV) terms (17 out of 27 respondents with sequence data) and QTL data with trait-related ontologies (eight out of 15 respondents with QTL data) ([Table 1](https://docs.google.com/document/d/1AuYfxqltINhp2VVeQNQ5_-gnNpo0aGClIjGsw6chdzo/edit#bookmark=id.f8ic9dq2bdov)). Trait-related ontologies were also used to curate germplasms (6 out of 16 respondents with germplasm data). Along with curation, respondents also reported using ontologies for data retrieval. Ten respondents answered that they use GO in gene search pages, and four respondents answered that they use trait-related ontologies in QTL search pages. Using ontologies to pull data from other data resources was not common (only four respondents). Most respondents noted that they intend increase use of ontologies in curation in the future, and nine respondents answered that they plan to incorporate ontologies such as the GO [[1]](https://paperpile.com/c/yG9wfR/OAbiV), TO [[2]](https://paperpile.com/c/yG9wfR/ihXJU) and PO [[3]](https://paperpile.com/c/yG9wfR/C00FU) in additional search pages. Two respondents answered that they intend to use ontologies to obtain data from other resources in the future.

Not all respondents reported using ontologies extensively for a variety of reasons. Barriers to using ontologies for curation included lack of resources including curator time, suitable tools for curation and validation, and ontologies that fit the domain or provide sufficient terms. Thus we identified a number of unmet needs for databases to support use of ontologies. These include:

1. Increased funding to train and pay biocurators
2. A tool or system where users can add terms and a timely review process for inclusion in the ontologies
3. Development of new ontologies that fit specific domains

we provide a review of currently available ontologies for agriculture and where to find them, a review of tools for working with ontologies, and a review of ontology curation practice in some databases.

**Supplementary material 2: How ontologies are used in agricultural and biological databases**

Ontologies are pivotal tools for standardizing data description. They enable integration and comparison of datasets within and across databases via shared terminology and semantics. Ontologies provide the semantic glue that links the idiosyncratic vocabularies developed for traits and other data important to each species. In this section, the ways ontologies are used in data descriptions in a sample of curated GGB databases are described.

**TAIR (**[**https://www.arabidopsis.org**](https://www.arabidopsis.org)**)** [[4]](https://paperpile.com/c/yG9wfR/OzrBZ)[[5]](https://paperpile.com/c/yG9wfR/hSra6): At TAIR, curators manually extract relevant experimental information about gene function from the research literature. The experimental results are codified in the form of GO and PO (expression) annotations. All GO and PO annotations (gene:term associations) are labeled with an evidence code (ECO; <http://www.evidenceontology.org>), a reference, and attribution to a source (i.e. the biocurator or community member who supplied the annotation). The GO ontologies are imported from the GO Consortium into TAIR’s internal curation tool on a monthly basis to ensure the latest versions are in use. In TAIR, GO and PO annotations are searchable by gene and keyword and are made available via quarterly data dumps and bulk download tools. GO annotations are exported in GAF2 format to the GO consortium. To ensure data accuracy and integrity, annotations are reviewed for quality control as part of the GO submission pipeline. TAIR has been curating GO annotations since 2001 and is the largest contributor of manual, literature-based annotations for *Arabidopsis thaliana*. Because many newly sequenced plant genomes rely on homology/orthology and domain architecture similarity for automated GO annotation, these experimentally based reference annotations are especially valuable for assigning and predicting functions of unknown genes.

**SoyBase (**[**https://www.soybase.org**](https://www.soybase.org)**)** [[6]](https://paperpile.com/c/yG9wfR/OlRy): Annotation of soybean QTLs (Quantitative Trait Loci) entails associating QTLs with PO and TO terms based on the description of the traits measured in the study. The description of most traits to which QTL have been measured are easily mapped to either PO and/or TO terms. In cases where the trait measured was the activity of an enzyme, it may also be possible to annotate the QTL with an Enzyme Commission (EC) number (www.brenda-enzymes.org) and Chemical Entities of Biological Interest (ChEBI) (<https://www.ebi.ac.uk/chebi>) terms for the enzymatic activity and products/reactants of the reaction catalyzed, respectively. Annotation of a soybean gene coding for an enzyme involves associating the enzymatic activity to one or more GO molecular function terms along with an EC number for the identification of the specific enzymatic activity. These genes can also be annotated with TO terms for the specific trait that the gene/activity has been associated with. In cases where definitive association with the gene expression and a specific plant tissue can be established, a PO anatomy term can also be assigned.

**GDR/CottonGEN/CSFL/CGD (**[**https://www.rosaceae.org**](https://www.rosaceae.org)**, https://www.bioinfo.wsu.edu)**: Gene models and transcripts in Genome Database for Rosaceae (GDR) [[7,8]](https://paperpile.com/c/yG9wfR/EODCY+L5A8e) are associated with GO terms by running InterProScan [[9]](https://paperpile.com/c/yG9wfR/UvGN1). QTL and heritable phenotypic markers are manually assigned to Rosaceae Trait Ontology (<https://www.rosaceae.org/trait_ontology>) which is an extended form of Plant TO. Each term in Rosaceae is either the same term as a TO term or has a parent TO term. QTL are also associated to PO, EC, ChEBI, or NCBI Taxonomy terms through associated TO. Genetic markers and gene models are also linked to Rosaceae TO terms when they are associated with the QTL or heritable phenotypic markers. CottonGEN [[10]](https://paperpile.com/c/yG9wfR/aFqXG), Cool Season Food Legume (CSFL), and CGD databases use crop-specific trait ontologies which are also extended forms of Plant TO.

**SGN/CassavaBase/SweetpotatoBase/YamBase/MusaBase (https://solgenomics.net)**: Germplasm in Sol Genomics Network (SGN)[[11]](https://paperpile.com/c/yG9wfR/z3yCC) are annotated with Solanaceae Phenotype Ontology (SPO) terms, describing mostly mutant phenotypes. Most of the Solanaceae ontology terms are mapped to PO and TO. Genes with experimental evidence are manually annotated using GO, PO, and also SPO terms if there is a known mutation. Gene models are annotated automatically with GO terms based on sequence similarities. In breeding experiments, individual plots or plants are measured using crop-specific ontologies. In addition to SPO for Solanaceae species (tomato, potato, pepper, eggplant, tobacco, petunia), other crop-specific trait ontologies include cassava, sweet potato, yam, and banana. Each ontology has a set of precomposed terms, which include the trait, method, and scale. This allows breeders to easily find the trait they need for measuring in field conditions. When using other ontologies to describe a trait, breeders can post-compose traits using for example ChEBI for a metabolite, PO for the plant part, Unit Ontology, and optional Time Ontology when a metabolite is measured at different times of the day.

**Gramene database (**[**http://www.gramene.org**](http://www.gramene.org)**)** [[12,13]](https://paperpile.com/c/yG9wfR/XX0DX+hy54A)**:** Gramene provides genome, gene function, synteny, genetic diversity data, Pathways and gene expression data at the Plant Kingdom level. The Gene Ontology (GO) is used to annotate gene function, biological processes and cellular location of a gene product. The proteins and enzymes function in pathways subscribe to ontologies from Uniprot and Planteome, and metabolites are cross-referenced to ChEBI. The Plant Ontology (PO) is used to describe both the anatomical structure where the gene is expressed or where the phenotype is observed, and the growth and development stage where gene expression or the phenotype is observed. The Plant Trait Ontology (TO) is used to annotate legacy cereal QTL traits and mutant phenotypes. The Plant Experimental Condition Ontology (PECO) is used to annotate the growth and conditions in which the gene expression or phenotypes were evaluated. The ontology-based data structure in Gramene and Plant Reactome allows complex synthesis of information for data discovery. A user can perform an integrated search from various portals, for example, to find information on individual genes, pathways, or traits, and then can navigate to a genome browser or paint a gene homology tree to find genes with common ontology annotations within or across multiple species (53 species in the genome portal and 75 species in the Plant Reactome portal).

**Animal QTLdb (**[**https://www.animalgenome.org/QTLdb**](https://www.animalgenome.org/QTLdb)**)**[[14]](https://paperpile.com/c/yG9wfR/SjbpX)**:** To accommodate the specialized traits used in livestock genetic studies, Animal QTLdb employs an internal trait hierarchy to which all QTL and SNP association data are annotated. The terms in the hierarchy are, in turn, manually annotated to corresponding terms in the Vertebrate Trait Ontology (VT; phenotypes related to morphology, physiology, or development [[15]](https://paperpile.com/c/yG9wfR/yBUyH)), Livestock Product Trait Ontology (LPT; phenotypes related to products obtained for human use and profit), and Clinical Measurement Ontology (CMO; morphological and physiological measurement records [[16]](https://paperpile.com/c/yG9wfR/YSfLB)).

**AgBase (**[**http://agbase.arizona.edu**](http://agbase.arizona.edu)**)** [[17]](https://paperpile.com/c/yG9wfR/Wi7m): AgBase curators manually extract relevant experimental information about gene function from livestock and aquaculture publications. The experimental results are codified in the form of GO annotations. We have also provided PO annotations for cotton based upon experimental data in published literature. GO annotations are submitted to the GO Consortium via the EBI GOA Project and PO annotations to the Plant Ontology Consortium via Gramene. For gene products where no literature exists we provide computational-based GO annotations based upon sequence analysis. All annotations are also available for download as GAF2 files via the AgBase website. AgBase also provides rapid, “first-pass” GO annotations to support functional analysis of non-model organisms with newly annotated genomes and transcriptomes (e.g., alligator, alpaca, Miscanthus, Daphnia, Cryptocercus).

**Supplementary material 3: Ontology resources**

In this section, we describe some of the available ontology libraries and registries where comprehensive lists of ontologies can be found.

The Planteome project (<http://planteome.org>) supports the development of reference ontologies for plants which are widely used throughout the plant genomics and agricultural database communities. These include the Plant Ontology (PO), Plant Trait Ontology (TO) and Plant Experimental Conditions Ontology (PECO), which are developed by the Planteome project, as well as other reference ontologies relevant for the plant community such as GO and ChEBI. The Planteome also hosts an extensive database of searchable and browsable ontology-based annotations for plant traits, phenotypes, and gene expression data across 95 plant species [[2]](https://paperpile.com/c/yG9wfR/ihXJU). The ontology terms, annotations and data entities are available through the Planteome web portal using AmiGO and via APIs. Planteome is included in the OBO Foundry (<http://www.obofoundry.org>)[[18]](https://paperpile.com/c/yG9wfR/RuumT) which is a reference community effort to help the biomedical and biological communities build ontologies with an enforcement of design principles and reuse that have made the effort very successful.

The Crop Ontology (CO) (www.cropontology.org) supports community development of crop-specific ontologies both by permitting requests for individual terms and by providing trait templates for CO terms [[19]](https://paperpile.com/c/yG9wfR/z0fwn). After submission and review by the group that manages the specific ontology, this template is converted to an ontology format using the CO API. In the trait template, a CO term is defined by a unique combination of a Trait, a Method, and a Scale. Currently, 21 crop-specific ontologies are available on the CO website and 10 of the CO ontologies for important crop plant species are integrated with the reference ontologies at Planteome by mapping each CO term to a related term in a reference ontology such as the Plant Trait Ontology (TO).

Ontology repositories that host agricultural ontologies include: the NCBO BioPortal (<http://bioportal.bioontology.org>)[[20]](https://paperpile.com/c/yG9wfR/HnQpw), OntoBee (<http://www.ontobee.org>)[[21]](https://paperpile.com/c/yG9wfR/8drpA), the EBI Ontology Lookup Service (<http://www.ebi.ac.uk/ols>) [[22]](https://paperpile.com/c/yG9wfR/GZQ0S), and AberOWL (<http://aber-owl.net>)[[23]](https://paperpile.com/c/yG9wfR/8UVWG). Each of these portals has been created to address the needs of the research community, starting from the core mission of hosting ontologies (browsing, searching, visualizing), to offering a mapping repository (store and generate alignments between ontologies), automatic data annotation service, ontology recommendation, community support, and reasoning capabilities.

The AgroPortal project (<http://agroportal.lirmm.fr>)[[24]](https://paperpile.com/c/yG9wfR/gEaDa) is an ontology repository for the agricultural domain built by reusing the openly available NCBO BioPortal technology [[25]](https://paperpile.com/c/yG9wfR/eN9yY). AgroPortal focuses on agriculture and is not limited to reference ontologies. AgroPortal also provides information about standards such as AGROVOC (a directory of agriculture and vocabulary) and the [National Agricultural Library](https://www.nal.usda.gov/) (NAL) Thesaurus, and the AnaEE Thesaurus. AgroPortal currently hosts 89 vocabularies or ontologies. In addition, 20 of the surveyed AgBioData database projects have been loaded and linked to their ontologies in AgroPortal. Users can choose a specific database project and explore the ontologies that the project uses (eg. <http://agroportal.lirmm.fr/projects/Planteome>). Users can also choose a specific ontology and see the list of projects that are using the ontology, which can be important for adoption and standardization of an ontology. Users can choose to view only the data from AgBioData community through a restricted user interface, at [http://agbiodata.agroportal.lirmm.fr](http://agbiodata.agroportal.lirmm.fr/).

AgroPortal also serves the Global Agricultural Concept Scheme (http://aims.fao.org/global_agricultural_concept_scheme_gacs), an initiative of the Food and Agricultural Organization of the United Nations (FAO), CAB International (CABI), and the National Agricultural Library of the US Department of Agriculture (NAL) to merge their agricultural thesauri: AGROVOC, CAB Thesaurus, and NAL Thesaurus. In the future, GACS will become a Concept Space, extended to map vocabularies and ontologies in the agriculture and food domains and will provide stable URIs for common concepts and their types. GACS aims to reduce the proliferation of one-to-one ontology alignments by offering a knowledge hub to which every semantic resources in agriculture and food can be attached ([http://agrisemantics.org/gacs)](http://agrisemantics.org/gacs) .

In addition to ontology portals, there are several “knowledge organization system” registries related to agriculture, such as FAIRsharing (<https://fairsharing.org>) [[26,27]](https://paperpile.com/c/yG9wfR/0j1tt+PkZ9w), the GODAN Action supported Map of Agri-food Data Standards (<http://vest.agrisemantics.org>, <https://f1000research.com/documents/7-177>) and the agINFRA linked data vocabularies (<http://vocabularies.aginfra.eu>) [[28]](https://paperpile.com/c/yG9wfR/FLIi). These services usually register ontologies and provide metadata as well as other resources, but do not support content-based services.

**Supplementary material 4. Tools for curating data with ontologies, editing ontologies, and annotation data exchange**

Respondents to our survey identified several factors that were perceived as limiting the use of ontologies by their databases. These factors included a lack of tools for curation and validation of annotations, and incomplete ontologies. In addition, respondents indicated a need for standardized data formats for data annotation and exchange. Significant work has been done in all of these areas; here we briefly review some of the available tools for data curation with ontologies and ontology editing, as well as data annotation and exchange formats ([Table 3](https://docs.google.com/document/d/1XjHdkT56p7ydAGvNYX7CsI7Z3ULTQW8sgOsmm7K-8V0/edit)).

Tools developed to facilitate the use of ontologies include software used exclusively by expert database biocurators to annotate database objects and community curation tools that allow subject experts (but not necessarily curators) to submit annotations. For example, curation tools for GO annotation include TAIR’s in-house curation tool PubSearch [[29]](https://paperpile.com/c/yG9wfR/4a1tL) and TAIR’s community portal the Online Annotation Submission Tool (TOAST) [[30]](https://paperpile.com/c/yG9wfR/EG83o), PomBase’s Canto [[31]](https://paperpile.com/c/yG9wfR/KPWrV) and the GO consortium’s Noctua ([http://noctua.berkeleybop.org](http://noctua.berkeleybop.org/)). To ensure data accuracy and quality, these curation tools generally incorporate pipelines that refresh the ontologies and carry out quality control steps to validate the annotations. For example, TAIR curators review all community curation submissions, and all submissions to GOC undergo automated quality control checks. An example of an automated check would be to ensure that a gene product is not accidentally associated to a term that is taxonomically restricted; e.g. a plant gene annotated to the term “cardiovascular system” would be flagged as an error.

**PubSearch** [[29]](https://paperpile.com/c/yG9wfR/4a1tL) is a literature curation tool that facilitates extraction of a variety of data types from published research including gene names, alleles, phenotypes, gene summaries, GO/PO annotations, and more. It is part of the “Generic Model Organism Database” or GMOD suite of tools and is designed for use by biocurators [[32]](https://paperpile.com/c/yG9wfR/qg08g). To address the challenge of insufficient curation resources, TAIR developed TOAST [[30]](https://paperpile.com/c/yG9wfR/EG83o) to encourage community contributions of GO and PO annotations and protein-protein interactions. TOAST has a simplified interface that restricts the selection of ontology terms and evidence codes based on annotation type and ontology. Annotations are then reviewed by expert curators from TAIR before integrating into the production database.

**Canto** (<http://gmod.org/wiki/Canto>)[[31]](https://paperpile.com/c/yG9wfR/KPWrV) is an open source web-based literature curation tool that was developed by PomBase that serves as both an in-house biocuration tool and community curation portal. It can be used to annotate gene function with GO terms and curate genetic and physical interactions. Ontology annotation data from Canto can be exported in Gene Annotation File Format (GAF). Canto is also a GMOD component that reuses other GMOD components including the Chado database schema.

**Noctua** (<http://noctua.berkeleybop.org>) is a web-based, open-source tool developed by the GO Consortium for collaboratively editing models of biological processes using ontologies. It enables curators to link GO annotations to other ontological annotations for the same entity, following a data model for causal ontology annotations known as the GO-CAM (Gene Ontology Causal Activity Models). To build biological models using ontology terms, Noctua can be used with a single ontology (e.g. GO) or multiple ontologies (e.g. GO with TO and/or PO at <http://noctua.planteome.org>). Noctua employs a reasoner on the back end, so it is possible to mix and match ‘pre-composition’ (single term) or ‘post-composition’ (multiple terms combined into a descriptive "sentence") approaches. For example, a trait term could be composed by adding a node for a quality from Phenotype and Trait Ontology (PATO) [[33,34]](https://paperpile.com/c/yG9wfR/yza1L+4ttai) connected to a node from PO or GO; alternatively a TO class can be used; the two will be inferred to be equivalent. Noctua allows simultaneous real-time editing by multiple curators. On the server side, it features the semantic representation of the data in triples enhanced with OWL (Ontology Web Language)[[35]](https://paperpile.com/c/yG9wfR/WqAtX) constructs for representing class expressions. Site-specific Noctua instances are available to registered users. It is currently in use by curators from Planteome, and the Gene Ontology Consortium.

**Table-Editor** (<http://bit.ly/table-editor>) is a lightweight web application that enables curators to easily edit data formatted as spreadsheets, while supporting autocomplete and selection from semantically associated ontologies and dictionaries. The YAML (YAML Ain't Markup Language) configuration in table-editor follows a lightweight standard for specifying OWL design patterns that can then be used for generating documentation, generating new terms, and retrofitting old ones, and provides autocomplete lookup as well as other assistance during the process of creating or editing portions of the knowledge graphs in a tabular format. The constrained and assisted table-editor ensures that the resulting data, in CSV or TSV format, are compatible with subsequent integration into a larger knowledge-base or ontology. As of the date of this publication, this application is still under development.

In the event that existing ontologies (some of which are described above) are insufficient or do not exist for a given knowledge domain, there needs to be a facile way to build and edit ontologies. A commonly used tool is Protégé (<http://protege.stanford.edu>)[[36]](https://paperpile.com/c/yG9wfR/fHUg0), a free, open-source platform that provides a suite of tools and applications for working with ontologies. Protégé’s plug-in architecture can be adapted to build both simple and complex ontology-based applications. Developers can integrate the output of Protégé with rule systems or other problem solvers to construct a wide range of intelligent systems.

To facilitate sharing annotations among resources, there are some existing and emergent standards for ontology annotation data exchange. The GO Annotation File (GAF) format is the standard for GO annotation data exchange (<http://geneontology.org/page/go-annotation-file-format-20>). Between the earlier GAF1.0 and GAF2.0 versions, additional fields were added to include contextual data about the annotation and the data types. GAF can be used for other types of gene-term annotations as well, and is used by some databases for sharing Plant Ontology (PO) gene expression annotations. Phenopackets (Phenotype Exchange Format or PFX; <https://github.com/phenopackets/phenopacket-format/wiki/Overview-of-Phenotype-Exchange-Format>) is an extensible data model and data exchange format for phenotype data from any species. It can be used with any phenotype ontology and can be extended with other ontologies such as quantity, environmental conditions, and evidence.

**References for Supplementary material 1, 2, 3, and 4**

1. [The Gene Ontology Consortium. Expansion of the Gene Ontology knowledgebase and resources. Nucleic Acids Res. 2016;45: D331–D338.](http://paperpile.com/b/yG9wfR/OAbiV)

2. [Cooper L, Meier A, Laporte M-A, Elser JL, Mungall C, Sinn BT, et al. The Planteome database: an integrated resource for reference ontologies, plant genomics and phenomics. Nucleic Acids Res. 2018;46: D1168–D1180.](http://paperpile.com/b/yG9wfR/ihXJU)

3. [Cooper L, Jaiswal P. The Plant Ontology: A Tool for Plant Genomics. Methods Mol Biol. 2016;1374: 89–114.](http://paperpile.com/b/yG9wfR/C00FU)

4. [Yoo D, Xu I, Berardini TZ, Rhee SY, Narayanasamy V, Twigger S. PubSearch and PubFetch: a simple management system for semiautomated retrieval and annotation of biological information from the literature. Curr Protoc Bioinformatics. 2006;Chapter 9: Unit9.7.](http://paperpile.com/b/yG9wfR/OzrBZ)

5. [Reiser L, Subramaniam S, Li D, Huala E. Using the Arabidopsis Information Resource (TAIR) to Find Information About Arabidopsis Genes. Curr Protoc Bioinformatics. 2017;60: 1.11.1–1.11.45.](http://paperpile.com/b/yG9wfR/hSra6)

6. [Grant D, Nelson RT. SoyBase: A Comprehensive Database for Soybean Genetic and Genomic Data. Compendium of Plant Genomes. 2017. pp. 193–211.](http://paperpile.com/b/yG9wfR/OlRy)

7. [Jung S, Lee T, Ficklin S, Yu J, Cheng C-H, Main D. Chado use case: storing genomic, genetic and breeding data of Rosaceae and Gossypium crops in Chado. Database . 2016;2016. doi:](http://paperpile.com/b/yG9wfR/EODCY)[10.1093/database/baw010](http://dx.doi.org/10.1093/database/baw010)

8. [Jung S, Ficklin SP, Lee T, Cheng C-H, Blenda A, Zheng P, et al. The Genome Database for Rosaceae (GDR): year 10 update. Nucleic Acids Res. 2014;42: D1237–44.](http://paperpile.com/b/yG9wfR/L5A8e)

9. [Jones P, Binns D, Chang H-Y, Fraser M, Li W, McAnulla C, et al. InterProScan 5: genome-scale protein function classification. Bioinformatics. 2014;30: 1236–1240.](http://paperpile.com/b/yG9wfR/UvGN1)

10. [Yu J, Jung S, Cheng C-H, Ficklin SP, Lee T, Zheng P, et al. CottonGen: a genomics, genetics and breeding database for cotton research. Nucleic Acids Res. 2014;42: D1229–36.](http://paperpile.com/b/yG9wfR/aFqXG)

11. [Fernandez-Pozo N, Menda N, Edwards JD, Saha S, Tecle IY, Strickler SR, et al. The Sol Genomics Network (SGN)—from genotype to phenotype to breeding. Nucleic Acids Res. 2014;43: D1036–D1041.](http://paperpile.com/b/yG9wfR/z3yCC)

12. [Tello-Ruiz MK, Naithani S, Stein JC, Gupta P, Campbell M, Olson A, et al. Gramene 2018: unifying comparative genomics and pathway resources for plant research. Nucleic Acids Res. 2018;46: D1181–D1189.](http://paperpile.com/b/yG9wfR/XX0DX)

13. [Naithani S, Preece J, D’Eustachio P, Gupta P, Amarasinghe V, Dharmawardhana PD, et al. Plant Reactome: a resource for plant pathways and comparative analysis. Nucleic Acids Res. 2017;45: D1029–D1039.](http://paperpile.com/b/yG9wfR/hy54A)

14. [Hu Z-L, Park CA, Reecy JM. Developmental progress and current status of the Animal QTLdb. Nucleic Acids Res. 2016;44: D827–33.](http://paperpile.com/b/yG9wfR/SjbpX)

15. [Park CA, Bello SM, Smith CL, Hu Z-L, Munzenmaier DH, Nigam R, et al. The Vertebrate Trait Ontology: a controlled vocabulary for the annotation of trait data across species. J Biomed Semantics. 2013;4: 13.](http://paperpile.com/b/yG9wfR/yBUyH)

16. [Smith JR, Park CA, Nigam R, Laulederkind SJ, Hayman GT, Wang S-J, et al. The clinical measurement, measurement method and experimental condition ontologies: expansion, improvements and new applications. J Biomed Semantics. 2013;4: 26.](http://paperpile.com/b/yG9wfR/YSfLB)

17. [McCarthy FM, Gresham CR, Buza TJ, Chouvarine P, Pillai LR, Kumar R, et al. AgBase: supporting functional modeling in agricultural organisms. Nucleic Acids Res. 2011;39: D497–506.](http://paperpile.com/b/yG9wfR/Wi7m)

18. [Smith B, Ashburner M, Rosse C, Bard J, Bug W, Ceusters W, et al. The OBO Foundry: coordinated evolution of ontologies to support biomedical data integration. Nat Biotechnol. 2007;25: 1251–1255.](http://paperpile.com/b/yG9wfR/RuumT)

19. [Shrestha R, Matteis L, Skofic M, Portugal A, McLaren G, Hyman G, et al. Bridging the phenotypic and genetic data useful for integrated breeding through a data annotation using the Crop Ontology developed by the crop communities of practice. Front Physiol. 2012;3: 326.](http://paperpile.com/b/yG9wfR/z0fwn)

20. [Salvadores M, Alexander PR, Musen MA, Noy NF. BioPortal as a Dataset of Linked Biomedical Ontologies and Terminologies in RDF. Semant Web. 2013;4: 277–284.](http://paperpile.com/b/yG9wfR/HnQpw)

21. [Ong E, Xiang Z, Zhao B, Liu Y, Lin Y, Zheng J, et al. Ontobee: A linked ontology data server to support ontology term dereferencing, linkage, query and integration. Nucleic Acids Res. 2017;45: D347–D352.](http://paperpile.com/b/yG9wfR/8drpA)

22. [Côté R, Reisinger F, Martens L, Barsnes H, Vizcaino JA, Hermjakob H. The Ontology Lookup Service: bigger and better. Nucleic Acids Res. 2010;38: W155–60.](http://paperpile.com/b/yG9wfR/GZQ0S)

23. [Slater L, Gkoutos GV, Schofield PN, Hoehndorf R. Using AberOWL for fast and scalable reasoning over BioPortal ontologies. J Biomed Semantics. 2016;7: 49.](http://paperpile.com/b/yG9wfR/8UVWG)

24. [Jonquet C, Toulet A, Arnaud E, Aubin S, Yeumo ED, Emonet V, et al. AgroPortal: A vocabulary and ontology repository for agronomy. Comput Electron Agric. 2018;144: 126–143.](http://paperpile.com/b/yG9wfR/gEaDa)

25. [Whetzel PL, NCBO Team. NCBO Technology: Powering semantically aware applications. J Biomed Semantics. 2013;4 Suppl 1: S8.](http://paperpile.com/b/yG9wfR/eN9yY)

26. [McQuilton P, Gonzalez-Beltran A, Rocca-Serra P, Thurston M, Lister A, Maguire E, et al. BioSharing: curated and crowd-sourced metadata standards, databases and data policies in the life sciences. Database . 2016;2016. doi:](http://paperpile.com/b/yG9wfR/0j1tt)[10.1093/database/baw075](http://dx.doi.org/10.1093/database/baw075)

27. [Sansone S-A, McQuilton P, Rocca-Serra P, Gonzalez-Beltran A, Izzo M, Lister A, et al. FAIRsharing: working with and for the community to describe and link data standards, repositories and policies [Internet]. 2018. doi:](http://paperpile.com/b/yG9wfR/PkZ9w)[10.1101/245183](http://dx.doi.org/10.1101/245183)

28. [Drakos A, Protonotarios V, Manouselis N. agINFRA: a research data hub for agriculture, food and the environment. F1000Res. 2015;4: 127.](http://paperpile.com/b/yG9wfR/FLIi)

29. [Yoo D, Xu I, Berardini TZ, Rhee SY, Narayanasamy V, Twigger S. PubSearch and PubFetch: a simple management system for semiautomated retrieval and annotation of biological information from the literature. Curr Protoc Bioinformatics. 2006;Chapter 9: Unit9.7.](http://paperpile.com/b/yG9wfR/4a1tL)

30. [Reiser L, Subramaniam S, Li D, Huala E. Using the Arabidopsis Information Resource (TAIR) to Find Information About Arabidopsis Genes. Curr Protoc Bioinformatics. 2017;60: 1.11.1–1.11.45.](http://paperpile.com/b/yG9wfR/EG83o)

31. [Rutherford KM, Harris MA, Lock A, Oliver SG, Wood V. Canto: an online tool for community literature curation. Bioinformatics. 2014;30: 1791–1792.](http://paperpile.com/b/yG9wfR/KPWrV)

32. [O’Connor BD, Day A, Cain S, Arnaiz O, Sperling L, Stein LD. GMODWeb: a web framework for the Generic Model Organism Database. Genome Biol. 2008;9: R102.](http://paperpile.com/b/yG9wfR/qg08g)

33. [Gkoutos GV, Green ECJ, Mallon A-M, Blake A, Greenaway S, Hancock JM, et al. Ontologies for the description of mouse phenotypes. Comp Funct Genomics. 2004;5: 545–551.](http://paperpile.com/b/yG9wfR/yza1L)

34. [Gkoutos GV, Schofield PN, Hoehndorf R. The anatomy of phenotype ontologies: principles, properties and applications. Brief Bioinform. 2017; doi:](http://paperpile.com/b/yG9wfR/4ttai)[10.1093/bib/bbx035](http://dx.doi.org/10.1093/bib/bbx035)

35. [Musen MA. Web Ontology Language (OWL). Encyclopedia of Systems Biology. 2013. pp. 2350–2351.](http://paperpile.com/b/yG9wfR/WqAtX)

36. [Musen MA, Protégé Team. The Protégé Project: A Look Back and a Look Forward. AI Matters. 2015;1: 4–12.](http://paperpile.com/b/yG9wfR/fHUg0)
